# Distinct morphometric features of cardiomyocytes isolated from mouse hypertrophy models: An ImageJ analysis combined with machine learning algorithms

**DOI:** 10.14814/phy2.70425

**Published:** 2025-06-19

**Authors:** Hoang Duc Minh Pham, Marie‐Thérèse Daher, Onnik Agbulut, Zhenlin Li, Ara Parlakian

**Affiliations:** ^1^ Sorbonne Université, CNRS UMR8263, INSERM U1345, Development, Adaptation and Ageing, Institute of Biology Paris‐Seine Paris France; ^2^ Present address: Center for Genetic Medicine Northwestern University, Feinberg School of Medicine Chicago Illinois USA

**Keywords:** cardiac hypertrophy, cardiomyocyte isolation, Langendorff, machine learning, morphological analysis

## Abstract

This study aims at defining a standardized workflow based on a customized ImageJ macro combined with a machine‐learning algorithm to analyze morphometric features of isolated cardiomyocytes using high‐resolution/high‐content photomicrographs and to identify key and specific morphological features of cardiomyocytes isolated from various murine cardiac hypertrophy models. For that purpose, we set up and optimized a Langendorff based protocol for isolating cardiomyocytes from mouse hearts. This optimized protocol yielded in a significantly high number of formaldehyde‐fixed cardiomyocytes, with more than 97% of rod shaped cells. Moreover, our method allowed for reliable gene expression analysis and conservation of cell integrity through multiple freeze–thaw cycles. Next, we successfully applied our analytical workflow on formaldehyde‐fixed cardiomyocytes isolated from various murine cardiac hypertrophy models and defined distinct morphological features in Angiotensin II, Isoproterenol, and age‐induced hypertrophy. Taken together, our study provides an effective and standardized workflow for high‐throughput morphological and molecular characterization of isolated cardiomyocytes, and could constitute a robust and reliable analytical tool to distinguish healthy versus diseased states and assess the ability of a potential therapeutic agent or strategy to reverse the situation.

## Graphical abstract

Key steps for the isolation and quantification process of cardiomyocytes.

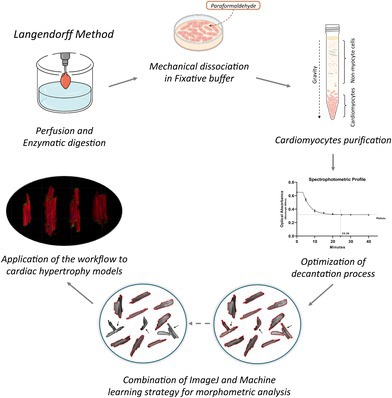

## 1 INTRODUCTION

Upon its stimulation by various physiological and pathological cues, the heart initiates a process called cardiac remodeling. This process is characterized by functional, molecular, metabolic, and morphological changes that can be either reversible (e.g., physiological hypertrophy) or irreversible (Eghbali et al., 2005; Dorn, 2007). In this latter case, the observed alterations can lead to heart failure and subsequent death (Dorn, 2007). Murine models of innate or acquired cardiomyopathies are extensively used to explore these changes and determine specific or common features for these pathologies (Gary‐Bobo et al., 2008; Hovhannisyan et al., 2024; Parlakian et al., 2005; Schrickel et al., 2010; Thornell et al., 1997). This can help or lead to the identification of potential therapeutic targets. Indeed, these models can be subsequently used to assess the beneficial impact of various therapeutic strategies in a preclinical setting (Deloux et al., 2017; Diguet et al., 2018; Domengé et al., 2021).

The careful and meticulous assessment of morphological features using high numbers of cardiomyocytes could constitute a robust and reliable method to distinguish healthy versus diseased states and assess the ability of a potential therapeutic agent or strategy to reverse the situation.

The 3D architecture of the mammalian heart is characterized by an intricate array of cardiomyocytes with both longitudinal and transverse orientations (Almasian et al., 2024). This arrangement of cardiomyocytes, makes it difficult to measure morphometric features such as length, width, cell surface, and volume on cardiac tissue sections with high numbers of cells (Bensley et al., 2016). As an alternative, several studies reported various strategies to isolate cardiomyocytes from murine or human hearts in order to determine such morphometric parameters (Ackers‐Johnson et al., 2016; Jian et al., 2016; Li et al., 2014, 2020; Liu et al., 2021; Louch et al., 2011; Nicks et al., 2022; Yücel et al., 2020; Zhou et al., 2022).

Over the last two decades, substantial improvement in optical components, motorized systems, and speed in image capture of microscopy devices allows the acquisition of high‐number/high‐quality data within a reasonable time frame. In parallel, the recently developed machine learning strategies and the availability of open source analytical tools with the possibility to modify/adapt the scripts for a given purpose allow the automated (semiautomated) treatment of high numbers of biological samples and the extraction of multiple features simultaneously.

In this study, we tested several cardiomyocyte isolation protocols and optimized isolation conditions that allowed us to recover intact rod‐shaped cardiomyocytes with high yield. Next, we used a combination of custom‐designed “Analyze‐Particle” based ImageJ macro and a supervised machine learning algorithm to analyze morphometric features of cardiomyocytes isolated from murine models of pathological (Angiotensin II or Isoproterenol induced) and age‐related hypertrophy (20‐month‐old mice).

## 2 METHODS

### 2.1 Animals

3‐ and 20‐month‐old (Old) C56BL/6 female mice were purchased from Charles Rivers and housed in a temperature‐ and humidity‐controlled facility with a 12‐h light/day cycle and given free access to standard rodent chow (#A03 Safe Diets) and water. For Angiotensin II (Ang II) and Isoproterenol (Iso) treatment, 3‐month‐old mice were subjected to a subcutaneously implanted osmotic minipump (Alzet model 1002, Charles Rivers, France) to continuously infuse either Ang II (#A9525, Sigma) at a dose of 3 mg/kg/day or Iso (#I6504, Sigma) at a dose of 40 mg/kg/day for a period of 14 days. All animal work was conducted in accordance with French regulations and experimental guidelines of the European Community, and the protocols were approved (N° #37927) by the local Animal Ethics Committee of Sorbonne University.

### 2.2 Mouse cardiomyocyte isolation

#### 2.2.1 Solutions and equipment setup

All the solutions (Table 1) were freshly prepared before the experiment. The circulating water bath connected to the Langendorff system (ADInstruments, France) was set to 37°C. Oxygen supply was maintained throughout the perfusion process. All surgical instruments were sterilized in preparation for the procedure. A cannula needle was attached to a syringe then filled with EDTA (#E8008, Sigma) buffer, ensuring no air is trapped within, and this assembly was then securely fastened to the dissecting microscope illuminator. A double loop 5–0 silk suture was loosely tied around the cannula needle, which will later secure the aorta. Two 60 mm petri dishes were filled with pre‐cold EDTA buffer and positioned one next to the surgical plate and the other under the dissecting microscope with the cannula tip submerged in the solution.

**TABLE 1:** Cardiomyocyte isolation solutions composition.

Wash buffer				
Reagent	Catalogue number, company	Final concentration (mM)	Molecular weight	Amount
NaCl	#S5886, Sigma	113	58.4	3.3 g
KCl	#P5405, Sigma	4.7	74.5	0.175 g
MgSO_4_	#M3409, Sigma	1.2	120.4	0.072 g
KH_2_PO_4_	#P5655, Sigma	0.6	136.09	0.041 g
NaH_2_PO_4_	#S5011, Sigma	0.6	119.98	0.036 g
HEPES	#H4034, Sigma	10	238	1.19 g
NaHCO_3_	#S6014, Sigma	1.6	84.01	0.067 g
BDM	#B0753, Sigma	10	101.1	0.505 g
Taurine	#T0625, Sigma	30	125.15	1.8775 g
Glucose	#G5500, Sigma	20	180	1.8 g
Milli‐Q water	Millipore	n/a	n/a	500 mL

Enzymatic digestion buffer			
Reagent	Catalogue number, company	Final concentration	Amount
Wash buffer		n/a	15 mL
Liberase™ Research Grade (26 U/mL)	5,401,119,001, Roche	0.433 U/mL	250 μL

Stop buffer (calcium reintroduction buffer 1)			
Reagent	Catalogue number, company	Final concentration	Amount
Wash buffer		n/a	20 mL
CaCl_2_ (1 M)	#21115, Sigma	0.2 mM	4 μL
BSA	#A8806, Sigma	5 mg/mL	100 mg

Calcium reintroduction buffer 2			
Reagent	Catalogue number, company	Final concentration	Amount
Wash buffer		n/a	20 mL
CaCl_2_ (1 M)	#21115, Sigma	0.5 mM	10 μL
BSA	#A8806, Sigma	5 mg/mL	100 mg

Calcium reintroduction buffer 3			
Reagent	Catalogue number, company	Final concentration	Amount
Wash buffer		n/a	20 mL
CaCl_2_ (1 M)	#21115, Sigma	1 mM	20 μL

EDTA buffer			
Reagent	Catalogue number, company	Final concentration	Amount
Wash buffer		n/a	99 mL
EDTA 100X	#E8008, Sigma	1 mM	1 mL

Fixative buffer			
Reagent	Catalogue number, company	Final concentration	Amount
Wash buffer		n/a	15 mL
Paraformaldehyde 8%	#P6148, Sigma	2%	5 mL

#### 2.2.2 Perfusion process

15 min before euthanasia, heparin (#H3393, Sigma) solution was injected intraperitoneally to the animal at a dose of 10 IU/g body weight. The mouse was anesthetized in a 5% isoflurane chamber for 5 min, then transferred to a cleaned surgical plate and maintained under anesthesia with a 4% isoflurane mask. Deep anesthesia was confirmed by the absence of reflex upon toe pinch. An incision was made to open the chest cavity and the heart was removed from the animal and immediately placed into a petri dish containing EDTA buffer to drain blood and to remove any noncardiac tissue. The heart was then transferred into a new petri dish. The cannula tip was carefully inserted into the aorta and secured with the silk suture. The perfusion system was filled with EDTA buffer to avoid any air in the system before connecting the cannulated heart to the Langendorff apparatus. The flow rate was set to 4 mL per minute, and the heart was perfused with EDTA buffer followed by Wash buffer for 3–4 min each, until the leak out solution become clear before performing downstream processes.

### 2.3 Ventricular cardiomyocytes isolation

“Standard Langendorff” protocol (SdL): The enzymatic digestion buffer was perfused through the heart for 18–22 min until the heart becomes soft, flaccid, and pale. The aorta and atria were trimmed. Ventricles were placed into a petri dish with Stop buffer and dissociated by pulling into 1 mm^3^ pieces, then pipetting gently with a Pasteur pipet. The suspension was passed through a 250 μm cell strainer (# 22363548, Thermo Fischer) to remove undigested fragments. The cells were subjected to three rounds of gravity settling with three intermediate calcium reintroduction buffers to bring back their physiological calcium concentration and to separate cardiomyocytes from non‐myocyte cardiac populations.

“Fixation prior to enzymatic digestion” protocol (FPED): the heart was perfused with Fixative buffer for 10–15 min followed by 15–30 min perfusion with wash buffer. The Enzymatic digestion buffer was circulated until the heart becomes digested. The subsequent steps were performed similar to the standard protocol.

“Dissociation in fixative buffer” protocol (DFB): The Enzymatic digestion buffer was perfused through the heart for 18–22 min until the heart becomes soft, flaccid, and pale. The aorta and atria were trimmed. Ventricles were quickly dipped into a petri dish containing Fixative buffer. The mechanical dissociation was performed by separating the ventricular tissue into eight parts with tweezers then pipetting with a Pasteur pipet and finishing with 1 mL pipet tip until cardiomyocytes are well dissociated. The suspension was filtered then transferred into a 15 mL falcon tube. Cardiomyocytes purification was done through several rounds of gravity decantation at 4°C in Fixative buffer for the first round and in PBS for subsequent decantation rounds. Purified cardiomyocytes were resuspended in BAMBanker™ freezing solution (#306‐95921, GCLymphotech Inc.) for further experiments.

### 2.4 Determination of the minimal time required for optimal gravity based decantation

The optical absorbance at 595 nm of the cell mixture in the supernatant was measured after 0, 5, 10, 15, 20, 25, 30 and 40 min, and then used to determine the “Plateau followed by one phase decay” using Prism (GraphPad) software. When 𝑌 falls within a 0.5% range of the Plateau value, it indicates that the Plateau has been effectively reached. The predicted time that indicates the end of cardiomyocyte stratification, was determined using the equation:

$$X_{t}=X_{0}-\frac{1}{k}.\ln\left(\frac{Y_{t}-\text{Plateau}}{Y_{0}-\text{Plateau}}\right)$$

Where,

𝑋_0_: the time at which the decay begins.

𝑌_0_: the initial value of 𝑌 at the reference point 𝑋_0_.

𝑌_𝑡_ the value of 𝑌 at a 0.5% range of Plateau.

Plateau: the value of 𝑌 at infinite times.

𝑘: the rate constant of decay.

### 2.5 RNA extraction from fixed cardiac cells

RNA was extracted from fixed cardiomyocytes using a modified protocol that preserves RNA integrity from paraformaldehyde (PFA) fixation. 5 × 10^5^ fixed cells were incubated in 100 μL of Uncross‐link buffer (Table 2) for 4 h at 55°C. RNA was then extracted using TRI Reagent® kit (#R2050‐1‐200 Zymo Research) and 1‐Bromo‐3‐chloropropane (#B62404, Sigma), following the manufacturer's instructions. The quality and quantity of the extracted RNA were evaluated using a NanoDrop 2000 spectrophotometer (#ND‐2000C, Thermo Scientific). The cDNA was prepared from 500 ng of total RNA using RevertAid RT Kit (#K1691, Thermo Fisher), according to the procedural guidelines. Quantitative real‐time polymerase chain reactions (qPCR) were run using GoTaq® qPCR Master kit (#A6002 Promega) in the LightCycler® 480 (Roche). The cycling conditions were set as previously described (Parlakian et al., 2005). Primer sequences are listed in Table S1.

**TABLE 2:** Uncross‐link buffer compositions.

Reagent	Catalogue number, company	Final concentration
NaCl	#S5886, Sigma	75 mM
SDS	#L3771, Sigma	1%
EDTA (pH 8.0)	#E8008, Sigma	2.5 mM
Proteinase K	#P2308, Sigma	6 mg/mL
RNase inhibitor	#AM2696, Invitrogen	1 U/μL

### 2.6 Immunofluorescence staining

For immunofluorescence staining, fixed‐cells were prepared at a concentration of 5 × 10^4^ cells in a 1.5 mL tube. Cells were suspended and incubated or not with permeabilization buffer (0.5% Triton X‐100 (#T8787, Sigma) in PBS) for 30 min followed by 30 min in blocking buffer (5% BSA (#A8806, Sigma), 0.1% Triton X‐100 in PBS) at room temperature. Cells were then incubated with primary antibodies in a 3% BSA solution for 3 h at room temperature or overnight at 4°C. Anti‐pan‐cadherin (Sigma; #C1821, dilution 1/250), anti‐α‐actinin (Sigma; #A7811, dilution 1/500), Alexa‐594 coupled Phalloidin (#A10256, Invitrogen; dilution 1/500), and Alexa‐488 coupled WGA (#11570806, Invitrogen; dilution 1/500) were used. The incubation with secondary antibodies was performed at room temperature for 1 h in the dark using either Alexa‐488 or 594 coupled mouse or rabbit IgGs (#A‐11001, or #A‐11005, Invitrogen, dilution 1/500). Cells were then incubated with Hoechst 33342 (#R37165, Invitrogen, 1:2000) to counterstain the nuclei for 5 min at room temperature. Swinging‐bucket centrifugation at 500 rpm for 3 min was used to wash and settle down cardiomyocytes and the supernatant was removed carefully by pipetting. For imaging, cell pellets were resuspended in Mowiol (#81381, Sigma) and 10 μL of the cell suspension was placed on a slide and covered with a 14 mm coverslip.

### 2.7 Image acquisition

Z‐stack images of 0.5–2 μm slice intervals were captured using a Leica K5 sCMOS microscope (Leica Microsystems, France) or a Leica TCS SP5 AOBS confocal microscope (Leica Microsystems, France) with a 20x/0.4 Dry or HCX Plan Apo 20×/0.7 objectives and Leica LAS AF software. For each sample, a total of 50 to 100 fields of view at a resolution of 2024 × 2024 pixels were captured for 2D image analysis. For volume measurement, a 2800μm^2^ area was randomly selected per sample.

### 2.8 Image analysis

3D images were obtained by combining Z‐stack images using the “Z project” function in ImageJ software (NIH, USA, version 1,54f). Image analysis was also performed on ImageJ using our “Analyze‐Particles” based macro for 2D analysis with the following script:

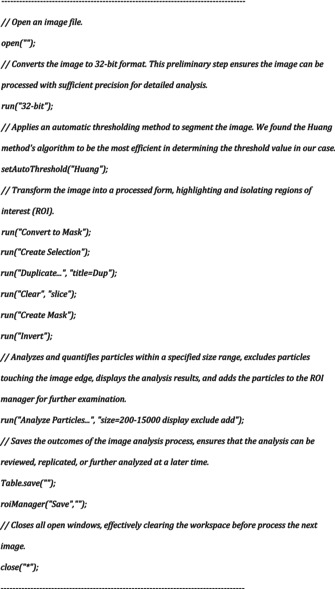

For volume measurement, “3D Objects Counter” and “3DSuite” was used. 3D images were generated using Arivis software (ZEISS).

### 2.9 Measurement classification

Data obtained from 2D image analysis was then further classified using the Pandas library (The pandas development team, 2024) and Logistic Regression (Sklearn), a machine‐learning algorithm. The non‐qualified samples were eliminated from the final calculations. The Python script was written and executed using Visual Studio Code (Microsoft, version 1.88).

Python script for the workflow was as follows:

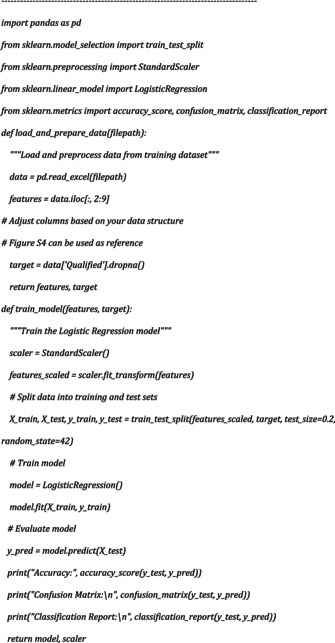

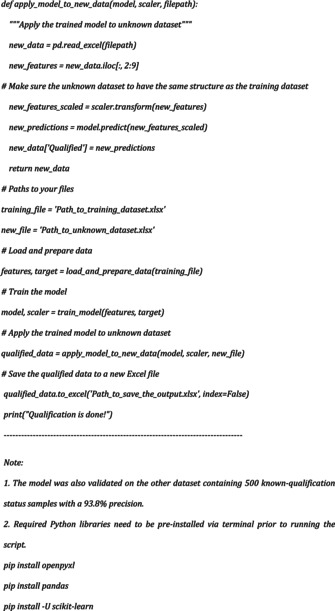

### 2.10 Data analysis

Statistical analyses were performed using GraphPad Prism (version 9.1) and RStudio (version 2024.9.0.375). Data are presented as mean ± SD. Differences between groups were analyzed using the Kruskal–Wallis test followed by Dunn's multiple comparisons test, one‐way ANOVA followed by Tukey's post hoc test, Mann–Whitney *U* test, or Fisher's exact test, as appropriate. Statistical significance was defined as *p* < 0.05.

## 3 RESULTS

### 3.1 Comparing the efficiency of various methods for isolating cardiomyocytes and optimization of the yield

**FIGURE 1:**
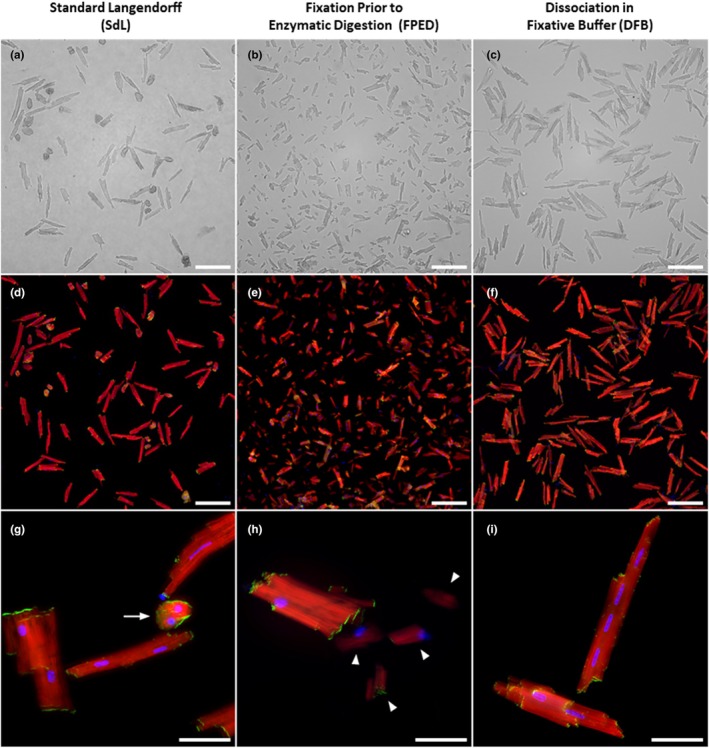
Comparative analysis of isolated cardiomyocytes under different methods. (a–c) Phase‐contrast images and (d–f) Fluorescence images of cardiomyocytes obtained using the standard Langendorff method (SdL), fixation prior to enzymatic digestion (FPED), and dissociation in fixative buffer (DFB). Cardiomyocytes were stained for actin (red), cadherin (green), and nuclei (blue). Scale bar: 100 μm. (g–i) Higher‐magnification fluorescence images highlight the structural details of individual cardiomyocytes. Arrows: Rounded cardiomyocyte. Arrowheads: Damaged and fragmented cardiomyocytes. Scale bar: 50 μm.

In order to isolate cardiomyocytes from adult murine hearts, we set up and compared the efficiency of three different Langendorff‐based methods as summarized in Table 3. The major difference between these methods was the step when fixation was performed. Indeed, for the first method designated as Standard Langendorff (SdL), the fixation was performed following enzymatic digestion and mechanical dissociation. For the second method designated as FPED (Fixation Prior to Enzymatic Digestion), fixation was performed by perfusion prior to the enzymatic digestion and mechanical dissociation. For the third method designated as DFB (Dissociation in Fixative Buffer), the fixation step was performed during the mechanical dissociation and after perfusion of the enzymatic solution. Following purification, we assessed the yield and integrity of cardiomyocytes based on multiple morphological criteria. We obtained the highest yield for cardiomyocyte cell number when using the DFB method (1.87 × 10^6^ ± 0.34 × 10^6^) as compared to the FPED (0.92 × 10^6^ ± 0.24 × 10^6^) and SdL (0.56 × 10^6^ ± 0.1 × 10^6^) methods, respectively, (Table 3). Using bright‐field photomicrographs (Figure 1a–c) and phalloidin/pan‐cadherin double immunostaining (Figure 1d–i), we assessed the percentage of intact rod‐shaped cardiomyocytes. An average of 97 ± 2% rod‐shaped cardiomyocytes was obtained when using the DFB protocol as compared to 70 ± 15% for the SdL and 50 ± 10% for the FPED methods, respectively, (Table 3 and Figure 1). Based on these criteria and additional morphometric parameters that we analyzed (described in Figures S1,S3 and Table 4), our results suggest that the DFB protocol is the most suitable for obtaining high yield and intact rod‐shaped cardiomyocytes.

**TABLE 3:** Overview of standard and modified Langendorff methods for cardiomyocyte isolation.

	Standard Langendorff (SdL)	Fixation prior to enzymatic digestion (FPED)	Dissociation in fixative buffer (DFB)
Step 1	Solutions preparation and equipment setup	Solutions preparation and equipment setup	Solutions preparation and equipment setup
Step 2	Anticoagulation and heart excision	Anticoagulation and heart excision	Anticoagulation and heart excision
Step 3	Cannulation and perfusion with wash buffer	Cannulation and perfusion with wash buffer	Cannulation and perfusion with wash buffer
Step 4	Enzymatic digestion by perfusion	Fixation by perfusion	Enzymatic digestion by perfusion
Step 5	Mechanical dissociation in stop buffer	Enzymatic digestion by perfusion	Mechanical dissociation in fixative buffer
Step 6	Filtration and purification	Mechanical dissection in stop buffer	Filtration and purification
Step 7	Fixation	Filtration and purification	

Outcome			
Integrity	Rod‐shaped cardiomyocytes: 70% ± 15% Rounded cardiomyocytes: 30% ± 15%	Rod‐shaped cardiomyocytes: 50% ± 10% Damaged and fragmented cardiomyocytes: 50% ± 10%	Rod‐shaped cardiomyocytes: 97% ± 2% Rounded cardiomyocytes: 3% ± 2%
Yield per Heart	Mean ± SD: 0.56 × 10^6^± 0.1 × 10^6^ (*n* = 15)	Mean ± SD: 0.92 × 10^6^ ± 0.24 × 10^6^ (*n* = 9)	Mean ± SD: 1.87 × 10^6^ ± 0.34 × 10^6^ (*n* = 20)

**TABLE 4:** Morphometrical comparison of isolated cardiomyocytes using standard and modified Langendorff methods. All data are expressed as mean ± SD.

	Standard Langendorff (SdL)	Fixation prior to enzymatic digestion (FPED)	Dissociation in fixative buffer (DFB)
Surface (μm^2^)	2482.36 ± 1329.69	1262.42 ± 1051.9	3117.42 ± 1324.12
Circularity	0.447 ± 0.184	0.617 ± 0.141	0.336 ± 0.093
Width (μm)	28.11 ± 8.93	24.29 ± 10.55	28.53 ± 8.62
Length (μm)	112.12 ± 46.61	56.96 ± 30.66	137.81 ± 31.78
Aspect ratio	2.39 ± 0.93	4.36 ± 2.27	5.18 ± 1.71

We were also able to show that the DFB method was suitable for gene expression analysis. Indeed, we successfully performed RT‐qPCR gene expression analysis on these cells. Following reverse transcription, we detected comparable Ct values for housekeeping genes (Figure S2) in PFA‐fixed versus non‐fixed cardiomyocytes. In addition, we found no significant difference in the expression level of cardiac specific genes (α‐MHC, β‐MHC, and α‐actin) under both conditions.

Given the high yield in cardiomyocyte cell numbers (1.87 × 10^6^ ± 0.34 × 10^6^), we tested also if cardiomyocytes can be stored at −80°C and sustain multiple freeze and thaw cycles without losing their morphology. We tested custom‐made and commercially available freezing solutions (DMSO based and BamBanker™ freezing solutions). Our results indicate that a majority of intact rod‐shaped cardiomyocytes with preserved sarcomere structure could still be observed after a four‐month freezing period in BamBanker™ solution (Figure S8).

### 3.2 Optimization of gravity based purification conditions

**FIGURE 2:**
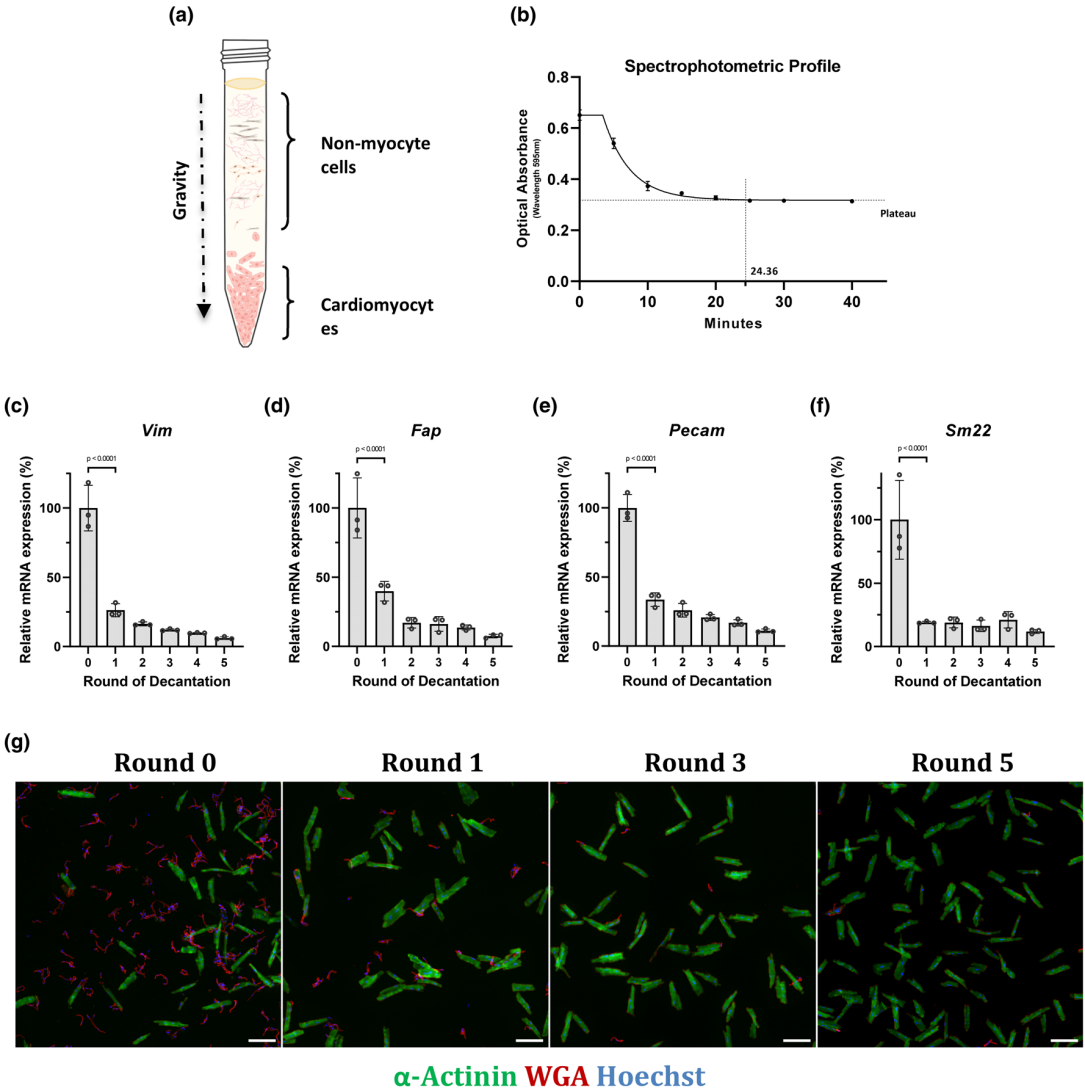
Optimization of the cardiomyocyte purification process using gravity decantation. (a) Schematic representation of cellular stratification in a 15‐mL falcon tube following gravity decantation, illustrating the separation of cardiomyocytes from non‐myocyte cells. (b) Kinetic spectrophotometric analysis of the cardiomyocyte purification process. The curve illustrates the “plateau followed by one phase decay” analysis. The plateau indicates the stabilization of cardiomyocyte stratification at 24.36 min (*n* = 3). (c–f) Relative mRNA expression levels of non‐myocyte markers: Vimentin, Fibroblast activation protein (FAP), Platelet endothelial cell adhesion molecule (PECAM), and Smooth muscle protein 22‐alpha (SM22), across five sequential rounds of decantation (*n* = 3). (g) Fluorescence images showing cardiomyocytes at various stages of decantation (0, 1, 3, and 5). Cells were stained with α‐Actinin (green), Wheat germ agglutinin (WGA) (red), and Hoechst (blue). The declining red staining serves as a measure of the decrease in non‐myocyte contamination. Scale bar: 100 μm. Data are expressed as mean ± SD. *p* values indicate statistical significance, with *p* < 0.05 considered significant.

Following this initial step of methodological optimization, we next sought to optimize purification conditions for the DFB protocol using gravity for selective cardiomyocyte decantation (Figure 2a). In order to determine the minimal time required for optimal cardiomyocyte decantation, we measured optical density (absorbance at 595 nm) of the cell mixture in the supernatant at time 0 and every 5 min during the decantation process. Using the profile of the exponential decay curve (Figure 2b) and a mathematical equation (described in Section 2), we determined that the minimal time required to reach a plateau was 24.36 min, which we rounded to 25 min. Next, to determine the number of decantation rounds that can be suitable for high content imaging approaches, we measured expression level of noncardiac markers in the cardiomyocyte enriched pellet after each round of decantation by RT‐qPCR. Results show that an average decrease of 73.57% for vimentin, 63.83% for FAP, 68.44% for PECAM, and 82.58% for Sm22 were observed following the first round of decantation (Figure 2c–f). After five rounds of decantation, the decrease in noncardiac gene expression was around 94.04% for vimentin, 93.26% for FAP, 88.69% for PECAM, and 89.73% for Sm22 (Figure 2c–f). We also monitored the depletion of noncardiac cells in the pellet by α–actinin/WGA double immunostaining. Qualitative assessment of the photomicrographs after each round of decantation suggests that three rounds of decantation are sufficient and compatible with high content image analysis (Figure 2g).

### 3.3 ImageJ‐based analysis combined with machine learning algorithms to define morphometric features of isolated murine cardiomyocytes

**FIGURE 3:**
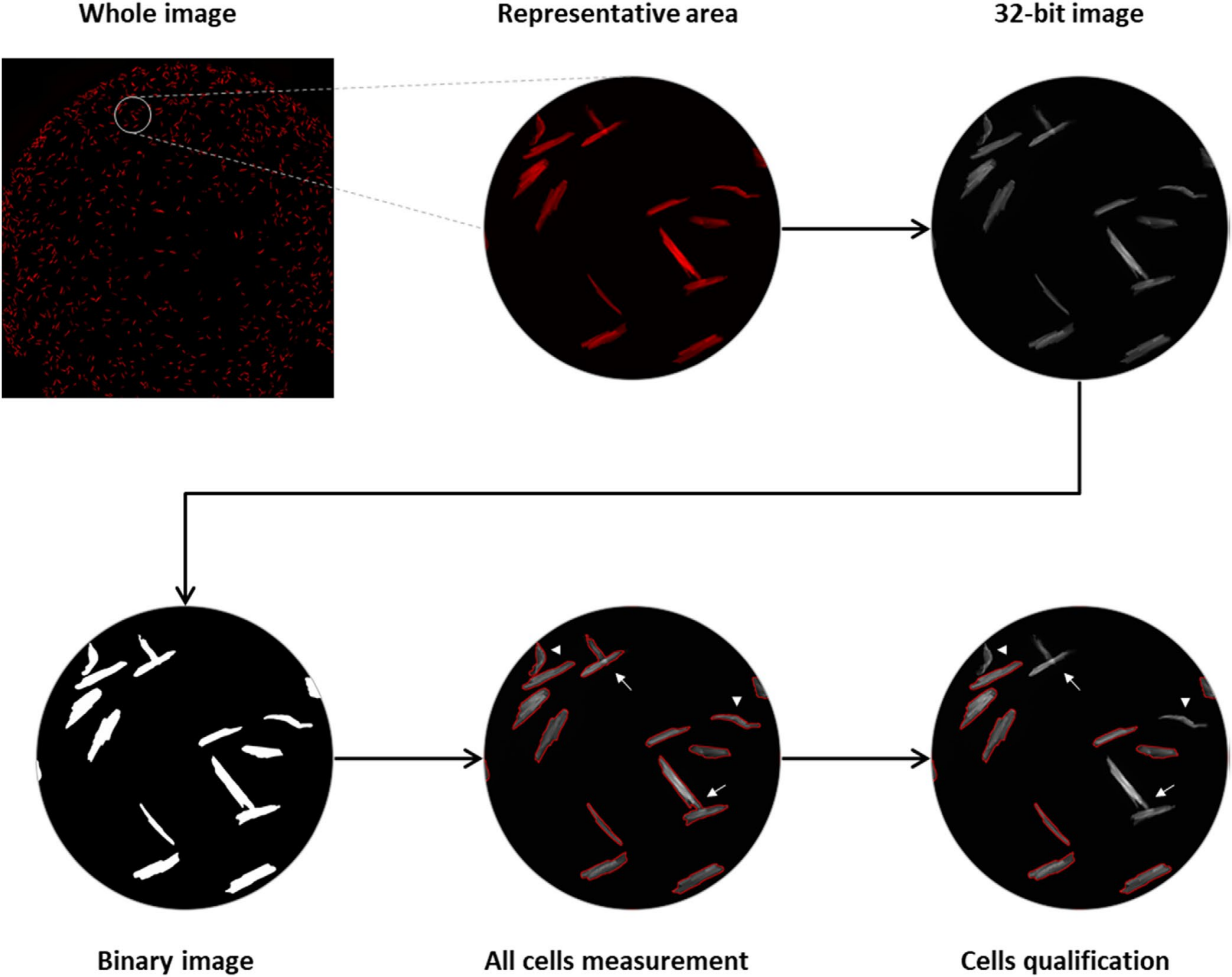
Representative workflow of morphometrical data generation and qualification. Cardiomyocytes were stained for Actin (red). Cells with a red outline indicate measured cells (all cells measurement) and qualified cells (cells qualification). The arrows indicate touching/overlaying cells. The arrowheads indicate cells with altered morphology.

In order to define morphometric features of cardiomyocytes isolated from mice in an accurate and automated manner using high resolution/high content imaging, we used a combination of a custom‐designed “Analyze‐Particle” based macro that relied on the built‐in functions of the ImageJ software and a supervised machine‐learning algorithm. The analytical workflow was performed in two main steps: morphometrical measurement and qualification of all cells (Figure 3). Cell size (surface), cell width, cell length, aspect ratio (AR) (Figure S3), and other parameters (perimeter, circularity, roundness, and solidity) were measured using a custom‐designed ImageJ macro (script described in Section 2).

While our custom‐designed macro was able to accurately detect and measure morphometric features of the cardiomyocytes, touching/overlaying or abnormally shaped cells present in the photomicrographs (Figure 3, arrows and arrowheads) could affect the outcome of the final results. In order to exclude such cases from the final results, we performed a data classification step utilizing Logistic Regression (Pedregosa et al., 2011), a supervised machine learning technique, to evaluate the qualification status of each measurement in the datasets. The model was trained using a training dataset consisting of 1000 cardiomyocytes with morphologically and visually verified qualification status. For each cell, eight morphometric parameters were considered: area, perimeter, circularity, AR, roundness, solidity, AR × Solidity, and scaled perimeter to area ratio (Figure S4). All parameters were normalized using the Standard Scale from “Sklearn” to ensure that each of them contributes equally to the model's decision and to prevent the dominance of features with greater values. Following the completion of the training procedure, the model underwent further validation using a distinct test set comprising 20% of the original dataset. Subsequently, this verified model was employed to categorize “unknown” datasets. A customized Python program (script described in Section 2) was utilized to manage the deployment, guaranteeing uniform pre‐processing and prediction methods across all datasets. The results were subsequently stored in Excel format, accompanied by indicators of whether the measurement was considered qualified (1) or not (0) (Figure S4). Only samples that met the necessary qualification status were utilized for the final computations. This method enabled the efficient batch processing of multiple datasets, as well as the convenient monitoring and evaluation of the model's predictions across different sample groups. Specifically designed for the accurate and consistent calculation of cardiac morphometric parameters, this tool allowed for automated analysis of large datasets (2600 to more than 5000 cardiomyocytes per condition) with up to 94% precision.

### 3.4 Morphometric features of cardiomyocytes in the context of cardiac hypertrophy

Following the setup of our workflow, we applied our isolation protocol in the context of cardiac remodeling in order to analyze and determine morphological changes of cardiomyocytes under pathological or age‐related hypertrophy. For that purpose, we used murine models of pathological hypertrophy (Ang II or Iso induced hypertrophy) and hypertrophic hearts of old animals (20‐month‐old mice). The cardiac remodeling process can be observed in aging animals or can be experimentally induced in rodents upon chronic vasoconstriction through the use of Ang II or chronic stimulation of β‐adrenergic receptors using Isoproterenol (Friddle et al., 2000; Wang et al., 2016). Molecular, metabolic, and morphological changes leading to hypertrophy under pathological conditions or during aging (Meschiari et al., 2017; Nakou et al., 2016; Shimizu & Minamino, 2016; Tracy et al., 2020) could differ based on the nature of signaling pathways and the chronology of events (Dorn, 2007).

**FIGURE 4:**
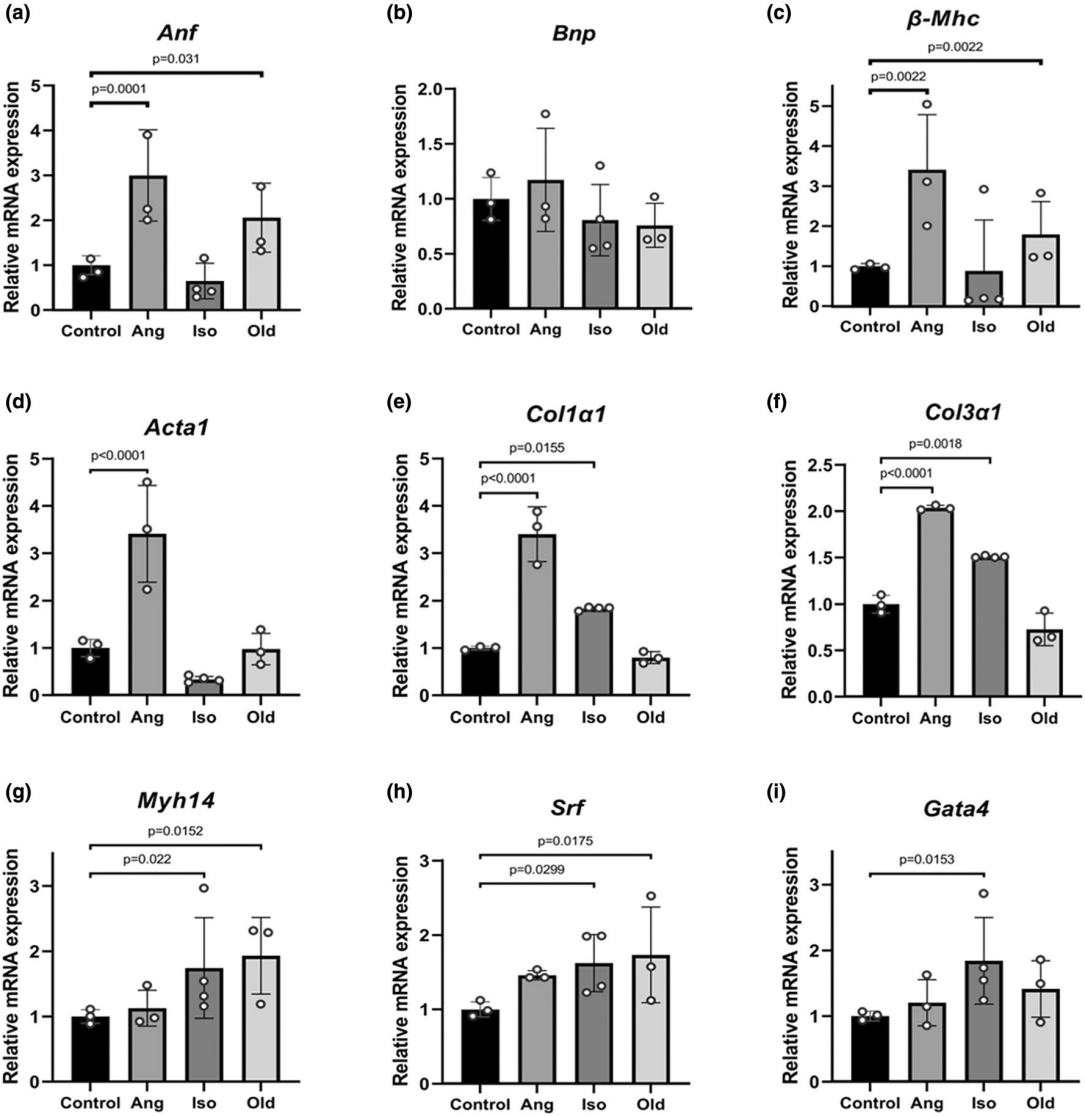
Differential gene expressions in cardiomyocytes subjected to various hypertrophic conditions. (a–i) Relative mRNA expression levels of hypertrophic and pre‐fibrosis markers: Atrial natriuretic factor (ANF), Brain natriuretic peptide (BNP), Beta myosin heavy chain (β‐MHC), Alpha skeletal muscle Actin (ACTA1), Collagen type I alpha 1 chain (COL1α1), Collagen type III alpha 1 chain (COL3α1), Myosin heavy chain 14 (MYH14), Serum response factor (SRF), and GATA binding protein 4 (GATA4) in cardiomyocytes under different treatment conditions: Control (*n* = 3), Angiotensin II (Ang) (*n* = 3), Isoproterenol (Iso) (*n* = 4), and Aging (Old) (*n* = 3). Data are expressed as mean ± SD. Points represent biological replicate means. Statistical significance was defined as *p* < 0.05.

In order to determine morphological and molecular features specific for each condition, optimized “DFB” protocol was used to isolate cardiomyocytes from control, Ang II‐treated, Iso‐treated, and Old mice. We first assessed the gene expression profile of isolated cardiomyocytes in control, Ang II‐treated, Iso‐treated, and Old mice. We found that the expression of stress and hypertrophy markers was significantly induced in all three groups with a differential pattern (Figure 4). More specifically, a significant increase in the expression of ANF and β–MHC was observed in the Ang II and old animal groups as compared to control (Figure 4a,c). Additionally, a significant increase in the expression of skeletal actin was observed in the Ang II‐treated group (Figure 4d). Interestingly, the expression of these genes did not show a significant increase in Iso‐treated cardiomyocytes, suggesting a different kinetic in their expression upon isoproterenol stimulation. However, and as expected, the expression of genes encoding for extracellular matrix components such as collagens I and III was induced in Iso‐ as well as Ang II‐treated cardiomyocytes (Figure 4e,f). The expression of Myh14, a marker of isoproterenol‐mediated hypertrophy (Wang et al., 2016), and the transcription factor SRF, a known mediator of cardiac hypertrophy, was significantly increased in Iso‐treated cardiomyocytes as well as in the cardiomyocytes of old animals (Figure 4g,h). Finally, the expression of GATA4, a cardiac‐specific transcription factor, was significantly increased only in Iso‐treated cardiomyocytes (Figure 4i).

Next, we assessed morphometric parameters for each group of cells and compared them to control. Cell size (surface), cell width, cell length, aspect ratio, and other parameters (perimeter, circularity, roundness, and solidity) were measured and qualified using the previously described workflow. Finally, cell volume assessment was also performed using 3D Objects Counter and 3D suite plugins (Ollion et al., 2013).

**FIGURE 5:**
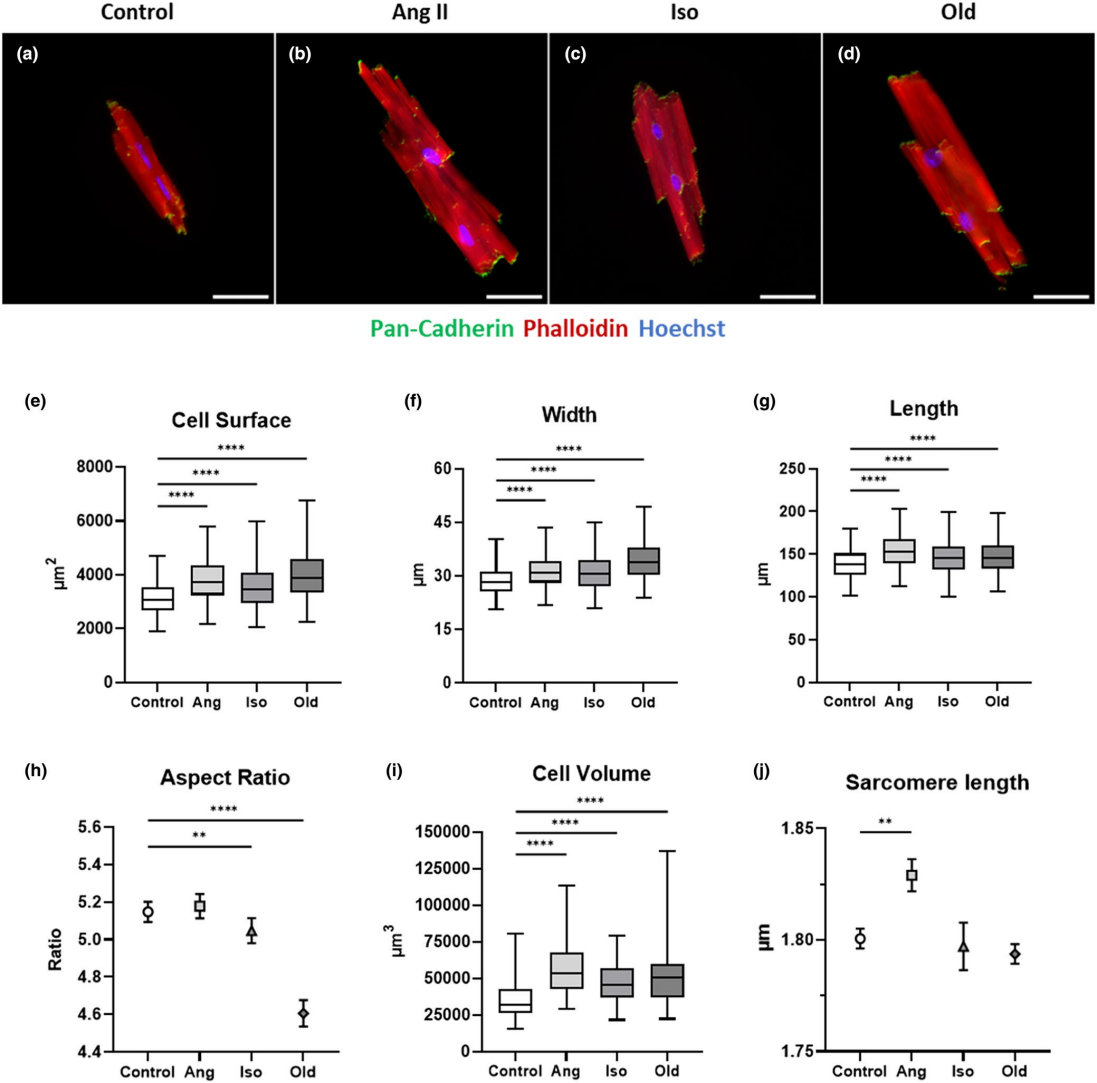
Morphological analysis of cardiomyocytes treated with four different conditions Control, Angiotensin II (Ang), Isoproterenol (Iso), and Aging (Old). (a–d) Representative fluorescence images of individual cardiomyocytes under the four conditions. The cells were stained for Actin (red), cadherin (green), and nuclei (blue). Scale bar: 50 μm (e–i) Quantifications of cell surface (e), cell width (f) cell length (g), aspect ratio (h) (Control: *N* = 3 animals, 5073 cells; Ang: *N* = 3 animals, 3311 cells; Iso: *N* = 3 animals, 3862 cells; Old: *N* = 3 animals, 2606 cells), and cell volume (i) (Control: *N* = 3 animals, 179 cells; Ang: *N* = 3 animals, 166 cells; Iso: *N* = 3 animals, 178 cells; Old: *N* = 3 animals, 135 cells). (j) Sarcomere length measurement (Control: *N* = 3 animals, 57 cells; Ang: *N* = 3 animals, 40 cells; Iso: *N* = 3 animals, 61 cells; Old: *N* = 3 animals, 50 cells). Data are presented as mean ± SD or box‐and‐whisker plots (boxes: Interquartile range with median; means shown as “+”; whiskers: 2.5th–97.5th Percentiles); *p* < 0.05 was considered statistically significant. **p<0.01; ****p<0.0001

As a result, a significant increase in cell size, width, length, and volume was detected in the three hypertrophic groups as compared to control (Figure 5, Figure S7, Table 5 and Video S1). Cell surface increased by 21.11%, 13.32%, and 27.25%, respectively, in cardiomyocytes of Ang II‐, Iso‐treated, and old animals (Figure 5e). These changes were the result of a proportionate increase in cell width (9.24%) and length (10.52%) for Ang II‐treated cardiomyocytes, while it was predominantly due to an increase in cell width for cardiomyocytes of the Iso‐treated and old animals' group. Indeed, cell width increased by 8.23% and 19.81%, respectively, in cardiomyocytes of Iso‐treated and old animals as compared to control (Figure 5f). As for the length, it increased only by 4.81% and 5.61%, respectively, for the cardiomyocytes of the Iso‐treated group and old mice group (Figure 5g). As a result, the aspect ratio remained unchanged for cardiomyocytes of Ang II‐treated animals, while it was significantly reduced in the Iso‐treated (−2.52%) and in the group of old animals (−10.85%) as compared to control (Figure 5h). Finally, cell volumes also increased in the three hypertrophic groups as compared to control. We observed a 60.93%, 38.24%, and 46.82% increase in cell volume, respectively, in Ang II‐, Iso‐treated, and cardiomyocytes of old animals (Figure 5i).

**TABLE 5:** Morphometrical comparison of cardiomyocytes treated under four different conditions: Control, Angiotensin II (Ang), Isoproterenol (Iso), and Aging (Old). All data are expressed as mean ± SD.

	Control	Ang	Iso	Old
Surface (μm^2^)	3169.79 ± 1244.09	3838.96 ± 1484.63	3592.17 ± 1428.37	4033.71 ± 1734.51
Width (μm)	28.78 ± 7.93	31.44 ± 8.29	31.15 ± 8.96	34.48 ± 10.44
Length (μm)	139.42 ± 33.06	154.09 ± 37.52	146.12 ± 34.29	147.24 ± 36.2
Aspect ratio	5.16 ± 1.71	5.18 ± 1.67	5.03 ± 1.72	4.6 ± 1.63
Volume (μm^3^)	35368.88 ± 19283.4	56919.39 ± 28243.97	48895.67 ± 21910.03	51928.17 ± 29308.98
Sarcomere length (μm)	1.801 ± 0.034	1.829 ± 0.045	1.797 ± 0.083	1.794 ± 0.031

We also measured the striation pattern of cardiomyocytes (Figure 5j and Figure SM1) in the four groups of mice and found a similar sarcomeric distance for Iso and old groups as compared to control (1.801 ± 0.004 μm in control vs. 1.797 ± 0.011 μm for Iso‐treated and 1.794 ± 0.004 for old group). Interestingly, a longer sarcomeric distance was observed for cardiomyocytes isolated from animals treated with Ang II (1.829 ± 0.007 μm) (*p* value: 0.0054).

Taken together, these results suggest that our DFB method could be successfully applied in pathological settings and generate a morphometric profile of the diseased heart based on multiple molecular and morphological features. Based on our analysis, we were also able to define a specific morphometric profile for each group.

## 4 DISCUSSION

In the current study, we compared and optimized a Langendorff‐based protocol to isolate and analyze fixed (non‐live) cardiomyocytes from adult murine hearts. We then successfully applied our isolation method on murine hearts undergoing pathological cardiac remodeling in response to Ang II and Iso. We also determined morphological features of cardiomyocytes in hypertrophic hearts of old animals. We also determined that dissociation of the ventricles in the fixative buffer was a key element in preserving the rod‐shaped morphology of cells and preventing the formation of round‐shaped or broken cardiomyocytes. This could be of critical importance when working on murine models of cardiomyopathies or hearts under ischemic conditions. Indeed, our isolation method would allow the inclusion of “fragile” cardiomyocytes for morphological analysis. Such cells could become round‐shaped or broken when using alternative isolation protocols and thus get excluded from analysis.

Using design‐based stereology of sections of heart tissue, Schipke J et al. have reported that the adult mice have 1.4–1.8 × 10^6^ cardiomyocytes (Werhahn et al., 2021), while Alkass K et al. have reported that cardiomyocyte number expansion is limited to the neonatal period and adult hearts of mice have 2.6 × 10^6^ cardiomyocytes (Perez‐Bonilla et al., 2024). The yield of rod‐shaped cardiomyocytes in our study (1.87 × 10^6^ ± 0.34) suggests that we were able to recover the majority number of ventricular cardiomyocytes present in the murine heart. The possibility to store PFA‐fixed cardiomyocytes at −80°C without losing their morphology (Figure S8) could be useful and convenient when performing time‐course analysis to monitor the progression of a given cardiac phenotype or to collect samples at different ages. Indeed, cardiomyocytes can be isolated at different time points, stored at −80 and processed for morphometric and/or molecular analysis simultaneously. In addition, this isolation process could be adapted and combined with various single‐cell/high‐throughput approaches such as single‐cell RNA‐seq or single‐cell ChIP‐seq analysis.

The expression of stress response marker such as ANF, BNP, and βHMC genes was not increased by Iso treatment in our system (Figure 4 and Figure S6). In order to validate our Iso‐induced hypertrophic model, we performed additional experiments by treating the mice with Iso‐containing osmotic mini‐pump during 14 days and assessed classical features of hypertrophy such as heart weight/body weight ratio and cross‐sectional area measurements. Indeed, the heart weight/body weight ratio and cross‐sectional area measurements confirmed the induction of hypertrophic changes in the heart of Iso‐treated mice (Figure S6). The expression of ANF, BNP, and βHMC gene were not increased in treated hearts as compared to control hearts thus confirming that the expression of these genes is not increased in our experimental conditions. Our results are consistent with that reported by several others (Perez‐Bonilla et al., 2024; Wang et al., 2016; Werhahn et al., 2021). Werhahn SM et al. used a new Iso on/off model to show the Janus‐headed nature of beta‐adrenergic signaling. They report that mice under constant Iso infusion during 3 weeks maintain their cardiac function, without overt changes in gene expression that are typically associated with heart failure. Withdrawal of Iso infusion resulted in the deterioration of cardiac function and activation of fetal and pathological genes (Werhahn et al., 2021). BNP together with ANF and β‐MHC is considered as a reliable marker of angiotensin II‐induced hypertrophy. While expression of both ANF and β‐MHC significantly increased, expression of BNP showed only a tendency to increase but did not reach statistical significance in our Ang II‐treated mice. Indeed, while most of the papers show an increase of BNP in Ang II treated mice, several papers have shown that BNP gene expression is not significantly increased by Ang II infusion, but tented to be increased as in our case (Matsumoto et al., 2013; Meyer Zu Schwabedissen et al., 2024).

Defining morphometric features of cardiomyocytes requires accurate measuring methods and is time‐consuming, especially when dealing with a large number of cells obtained after the isolation process. Based on a combination of custom‐designed ImageJ macros and Logistic Regression machine learning approaches, we were able to determine specific morphometric features using a relatively high number of cardiomyocytes (ranging from 2600 to 5000 cells) within a short time frame (less than an hour for processing an average of 10 photomicrographs containing 200–500 qualified cells each). This strategy was selected based on its robust performance in binary classification tasks, ease of implementation, and scalability, with no limitations on the number of cardiomyocytes that can be processed. The model achieved an accuracy, precision, and recall of approximately 94%, with an F1‐score of 93%, underscoring its effectiveness in distinguishing between different cell categories.

ImageJ is widely recognized in the scientific community, particularly in fields related to cell biology research and other disciplines, as a tool for image processing and analysis. However, Python, while popular in data science and machine learning, is not as commonly associated with biological image analysis. This suggests a promising opportunity for integrating the Logistic Regression based morphometric analysis approach into ImageJ as a plugin or module. Doing so would allow researchers, who are familiar with ImageJ, to easily access advanced machine learning techniques without requiring proficiency in Python or external programming libraries. Indeed, the analytical workflow described in our study illustrates the efficiency and usefulness of such a strategy.

Finally, we implemented and validated our isolation process and analytical workflow in the context of cardiac remodeling and more specifically in the context of acute pathological hypertrophy (Ang II and Iso stimulated hearts) and chronic hypertrophy (age related). While these models are commonly used in cardiac biology and the molecular features related to each signaling pathway are relatively well defined (Khalilimeybodi et al., 2020), comparative studies to highlight morphological features using high numbers of cell and specific for each type of hypertrophy in adult murine cardiomyocytes are lacking. Indeed, we were able to define specific morphometric features for each type of hypertrophy based on our custom‐made analytical tools. Our data suggest that hypertrophy is mainly due to an increase in cardiomyocyte cell width in Iso‐mediated (8.23%) or age‐related (19.81%) hypertrophy; while Ang II‐mediated hypertrophy is the result of a proportionate increase in cell width (9.24%) and length (10.52%). Cardiomyocyte hypertrophy correlate directly with sarcomeric addition. Yang H et al. have observed three ways of sarcomeric addition during cardiomyocyte hypertrophy. They include (1) insertion in the middle or addition at the end of a myofibril; (2) serial addition at the side using an existing myofibril as a template; and (3) longitudinal splitting of an existing myofibril (Yang et al., 2016). Ang II‐mediated hypertrophy could involve the three modes of addition, while Iso‐mediated or age‐related hypertrophy could use preferentially the mode of serial addition and/or longitudinal splitting. Furthermore, the increase in cell length in the Ang II‐treated group could be in part related to a longer sarcomeric distance. Indeed, several studies suggest that Ang II could regulate pre‐mRNA splicing and more specifically influence Titin splicing variants that are critical for sarcomere length (Cai et al., 2019; Joumaa et al., 2018; Loescher et al., 2022). Due to the use of fixative, our isolation method has some limitations. Indeed our method is not compatible with cell culture and do not allow proper extraction of proteins for biochemical analysis.

Taken together, the results of this study demonstrate that dissociation of the digested heart in a fixative buffer allows the efficient recovery of high numbers of intact rod‐shaped cardiomyocytes for morphological characterization and is compatible with gene expression analysis. We also showed that our optimized protocol could be applied to pathological and physiological models of cardiac hypertrophy. Finally, the standardized workflow, based on a customized ImageJ macro combined with a machine‐learning algorithm, that we developed can be applied on multiple settings and help compare healthy versus diseased states as well as assess the efficacy of therapeutic strategies in preclinical murine models of cardiomyopathies based on morphological criteria.

## FUNDING INFORMATION

This work was supported in part by Sorbonne University, INSERM, and CNRS. Zhenlin Li and Ara Parlakian are supported by the AFM‐Téléthon (contract number: 17689), Fondation de France (contract number: 00086490), the Fédération Française de Cardiologie (contract number: FFC2019), Emergence grant from Sorbonne University (EMERG‐28), and Fondation de l'Avenir (contract number: AP‐RM‐23‐028). Marie‐Thérèse Daher was the recipient of an AFM‐Telethon fellowship for her Ph.D. (Grant Numbers: 19353 and 22081). Hoang Duc Minh Pham was the recipient of the Vingroup Science and Technology Scholarship program for his Ph.D. (Cohort 2020).

## ETHICS STATEMENT

All animal work was conducted in accordance with French regulations and experimental guidelines of the European Community, and the protocols were approved (No. #37927) by the local Animal Ethics Committee of the Sorbonne University.

## Supporting information


Appendix S1.

Video S1.

## Data Availability

All data will be available upon publication of the manuscript, the ImageJ macro and the python script to execute the analysis are included in the text.
