# Supplementary material for: Distinct morphometric features of cardiomyocytes isolated from mouse hypertrophy models: An ImageJ analysis combined with machine learning algorithms
Source: Physiol Rep. 2025 Jun 19;13(12):e70425. doi: 10.14814/phy2.70425 (PMC12179408; doi:10.14814/phy2.70425)
Supplement: Supplementary file 1 — Appendix S1. [file PHY2-13-e70425-s001.docx]

**Supplemental Material**

**Distinct morphometric features of cardiomyocytes isolated from mouse hypertrophy models: An ImageJ analysis combined with machine learning algorithms**

**Hoang Duc Minh Pham^1^, Marie-Thérèse Daher^1, a^, Onnik Agbulut^1^, Zhenlin Li^1*^, Ara Parlakian^1*^**

^1^ Sorbonne Université, CNRS UMR8263, INSERM U1345, Development, Adaptation and Ageing, Institute of Biology Paris-Seine, Paris, France.

^a^ Present address: Center for Genetic Medicine, Northwestern University, Feinberg School of Medicine, Chicago, IL.

^*^ Corresponding authors

Corresponding authors:

Ara Parlakian (Tel: +33(0)144273128 Email: ara.parlakian@sorbonne-universite.fr)

Li Zhenlin (Tel : +33(0)144272136 Email: zhenlin.li@sorbonne-universite.fr)

Sorbonne Université, CNRS UMR8263, INSERM U1345, Development, Adaptation and Ageing, Institute of Biology Paris-Seine, Paris, France.7 quai Saint Bernard, 75 252 PARIS CEDEX, France.

**A**

**B**

**C**

**D**

**Figure S1.** Cell surface **(A)**, cell circularity **(B)**, cell width **(C)** and **(D)** aspect ratio analysis of isolated cardiomyocytes using standard Langendorff (SdL), fixation prior to enzymatic digestion (FPED) and dissociation in fixative buffer (DFB) methods. (SdL: N = 3 animals, 163 cells; FPED: N = 3 animals, 282 cells; DFB: N = 3 animals, 297 cells). Data are expressed as mean ± SD and frequency distribution. p-values indicate statistical significance, with p < 0.05 considered significant.


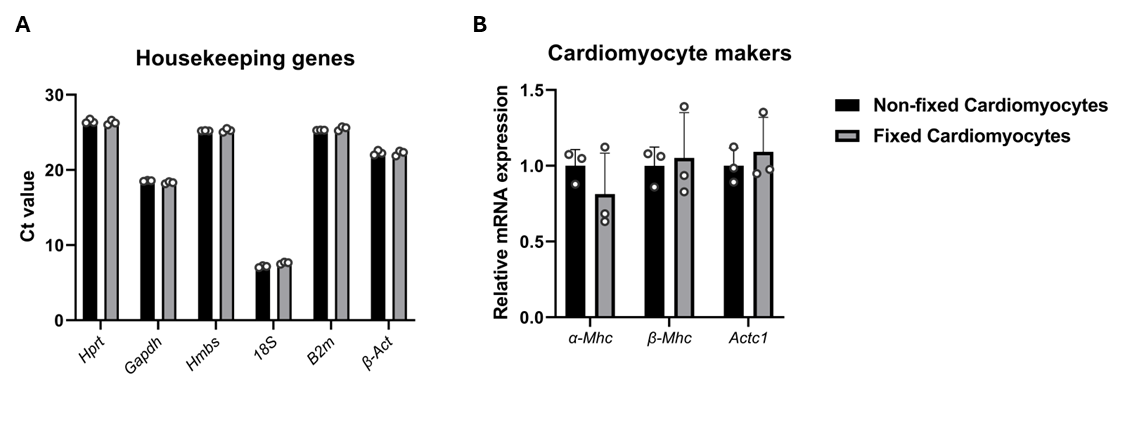


**Figure S2.** Expression profiles comparison of housekeeping and cardiomyocyte-specific genes between non-fixed (live) and fixed cardiomyocytes. **(A)** Cycle threshold (Ct) values for a selection of housekeeping genes: Hypoxanthine phosphoribosyl transferase (HPRT), Glyceraldehyde 3-phosphate dehydrogenase (GAPDH), Hydroxymethylbilane synthase (HMBS), 18S ribosomal RNA (18S), Beta-2 microglobulin (B2M), and Beta-Actin (β-Actin). **(B)** The relative mRNA expression levels of cardiomyocyte markers: Alpha myosin heavy chain (α-MHC), Beta myosin heavy chain (β-MHC), and Cardiac actin (c-Actin). These profiles show the consistent expression of examined genes across samples. N = 3 animals. All data are expressed as mean ± SD.


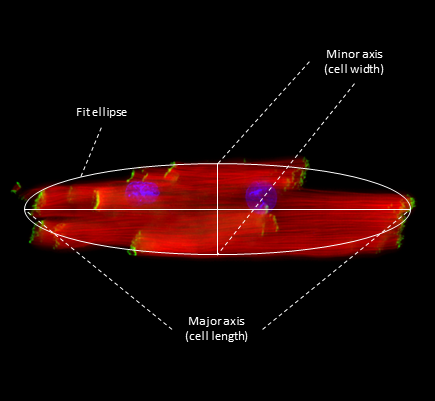


**Figure S3.** Ellipse fitting of a cardiomyocyte image was determined using ImageJ software.

In our experiment, the minor axis and major axis of a cardiomyocyte’s fit ellipse were considered as cell width and cell length, respectively. The aspect ratio was calculated as below:

$Aspect ratio = \frac{Major axis}{Minor axis}$


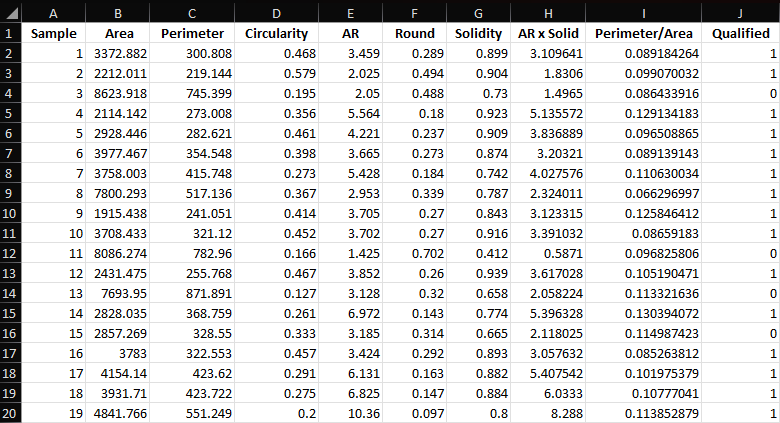


**Figure S4.** Representative table of morphometric data imported into Microsoft Excel software. Eight parameters (area, perimeter, circularity, aspect ratio, roundness, solidity, aspect ratio x solidity, and scaled perimeter to area ratio) were used for classification. The “Qualified” column indicates whether a sample is qualified (1) or not (0). Circularity reflects the smoothness of the cell’s boundary by comparing its perimeter to its area, while roundness measures how elongated the cell is along its major axis. During the ImageJ step, we applied an area threshold (200 - 15,000 µm²) to exclude small debris and large aggregates. During Logistic Regression classification, no explicit thresholds were applied to other morphometric parameters. Each cardiomyocyte was qualified based on the input features, rather than on preset morphometric cutoffs.


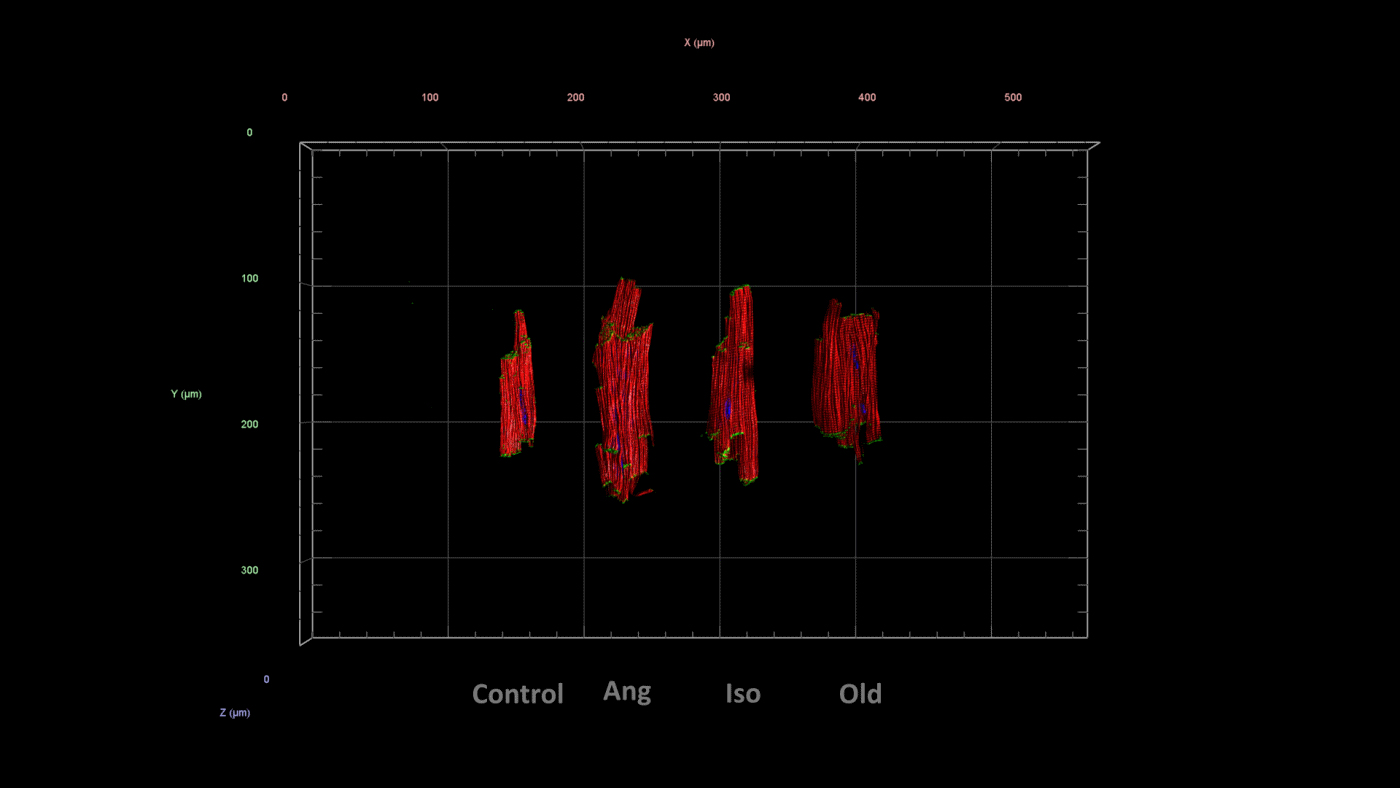


**Figure S5.** Representative 3D reconstructions of cardiomyocytes treated under four different conditions: Control, Angiotensin II (Ang), Isoproterenol (Iso), and Aging (Old). Cardiomyocytes were stained for actin (red), cadherin (green), and nuclei (blue). A 360-degree 3D composite image illustrates the spatial organization and morphology of cardiomyocytes. The X, Y, and Z axes represent spatial dimensions in micrometers. This image provides a comparative visual analysis of cardiomyocyte dimensions and architectural changes induced by hypertrophic stimuli and aging.

**Control**

**Iso**

**Phalloidin WGA Dapi**

**A**

**K**

**L**

**B**

**C**

**D**

**E**

**F**

**G**

**H**

**I**

**J**

**M**


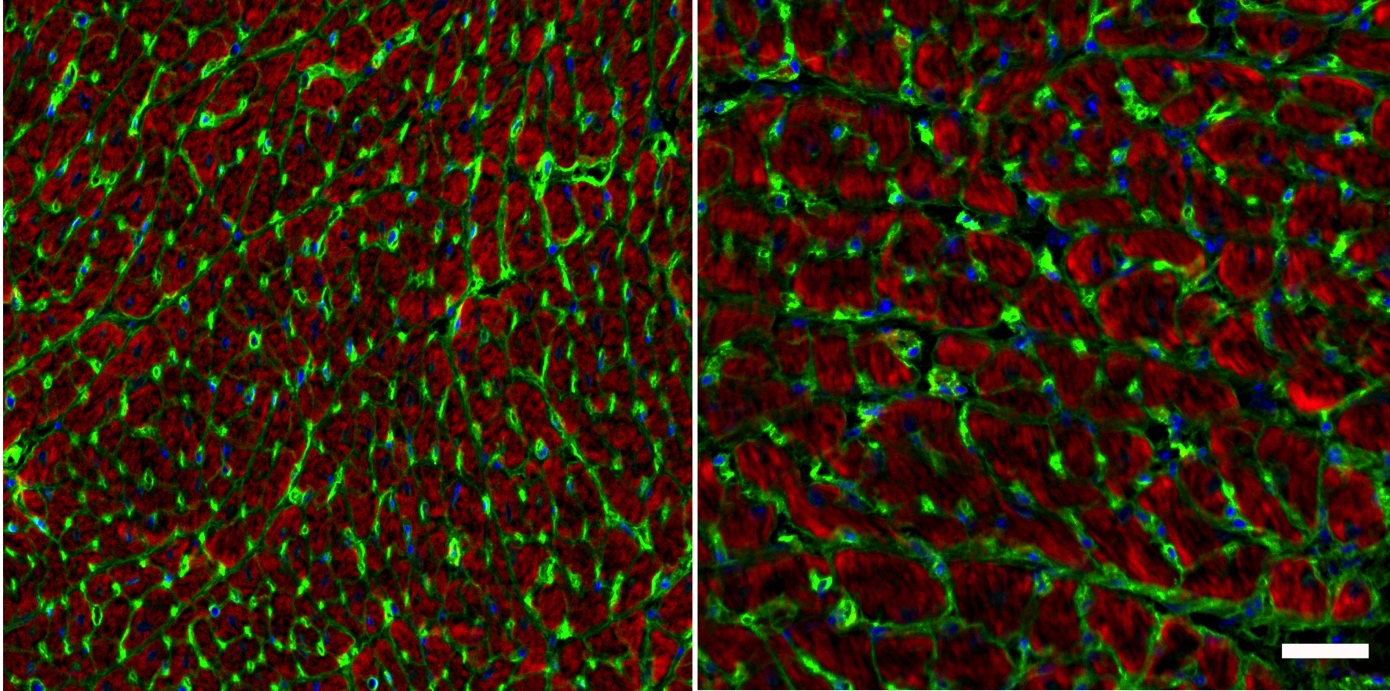


40μm

40μm

**Figure S6**. Morphological and molecular features of Iso-induced cardiac hypertrophy

A) Representative images of control and Iso-treated heart sections labelled with Phalloidin, WGA and Hoechst. B) Heart/Body weight ratio, C) Cross-sectional area measure and D) Min Feret. E-M) RT-qPCR gene expression analysis. Statistical comparisons were performed using Mann-Whitney test, with p < 0.05 considered significant


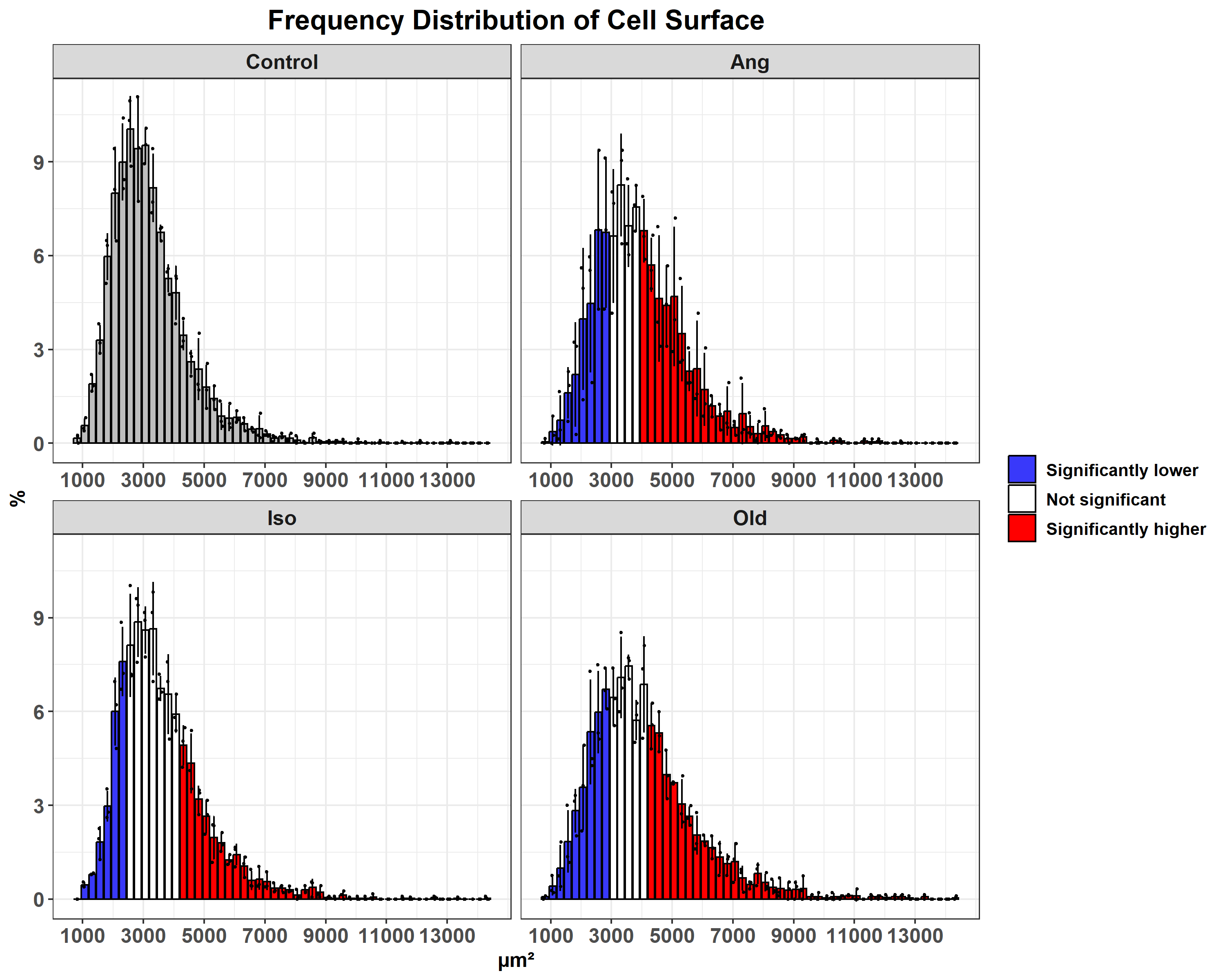

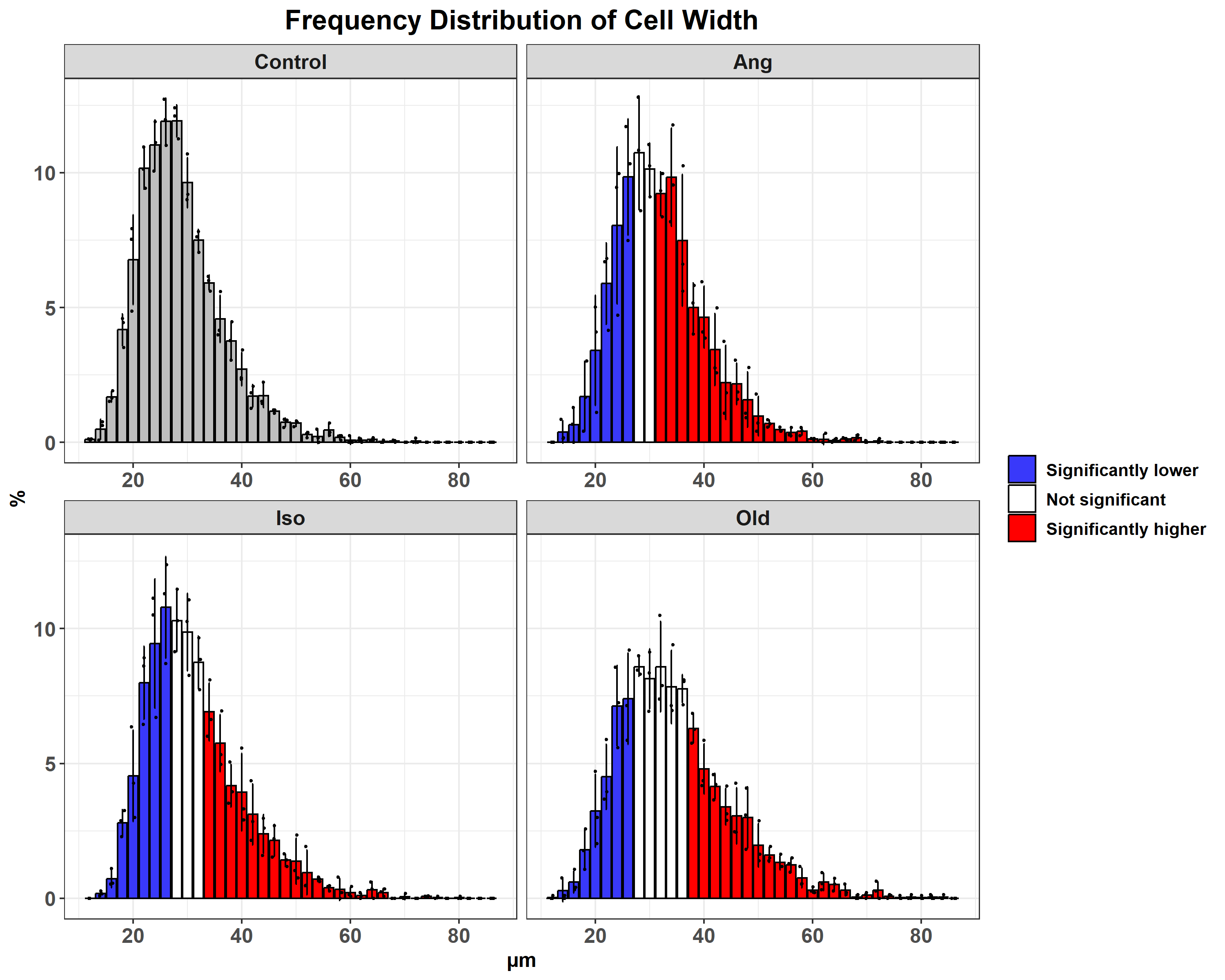

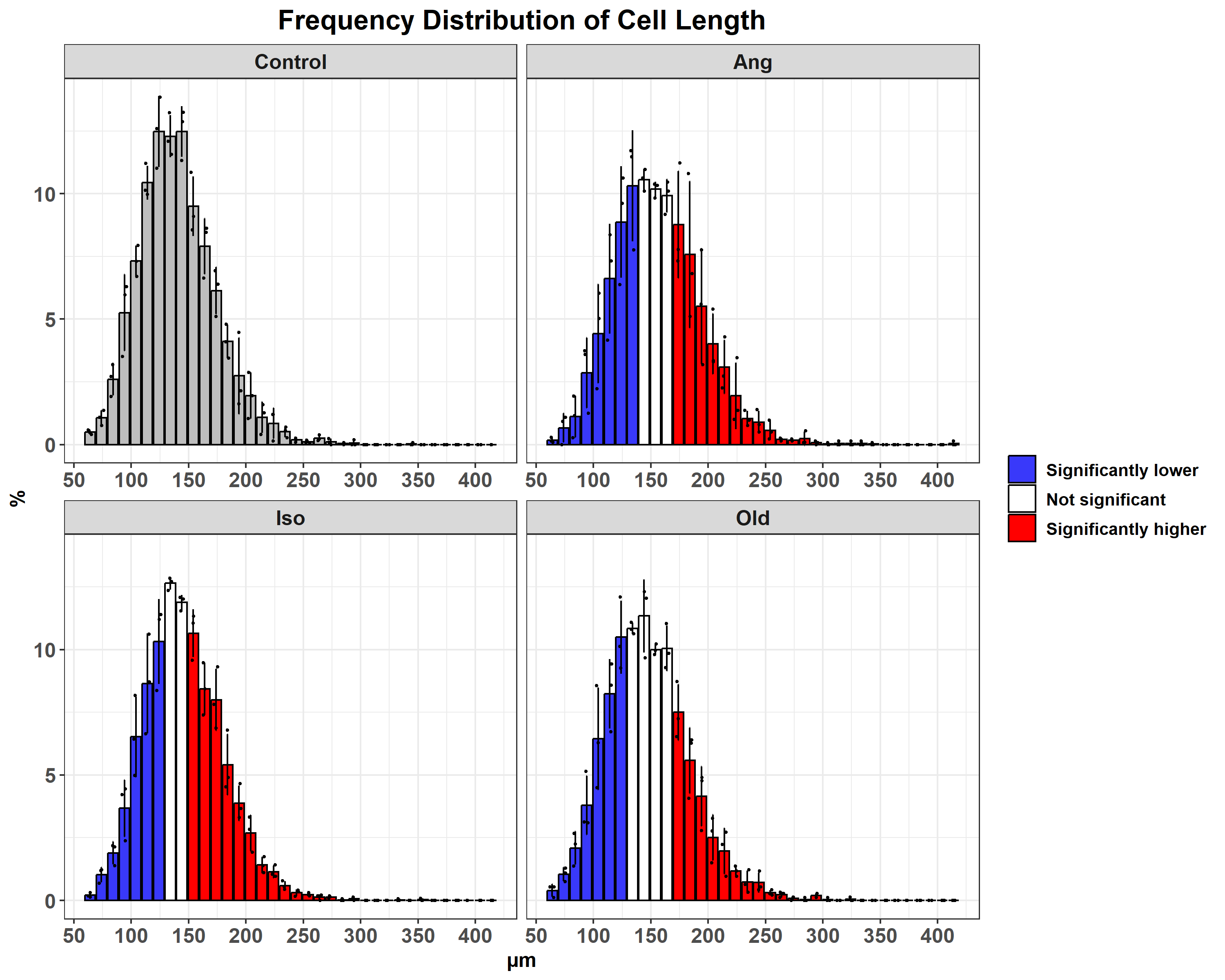

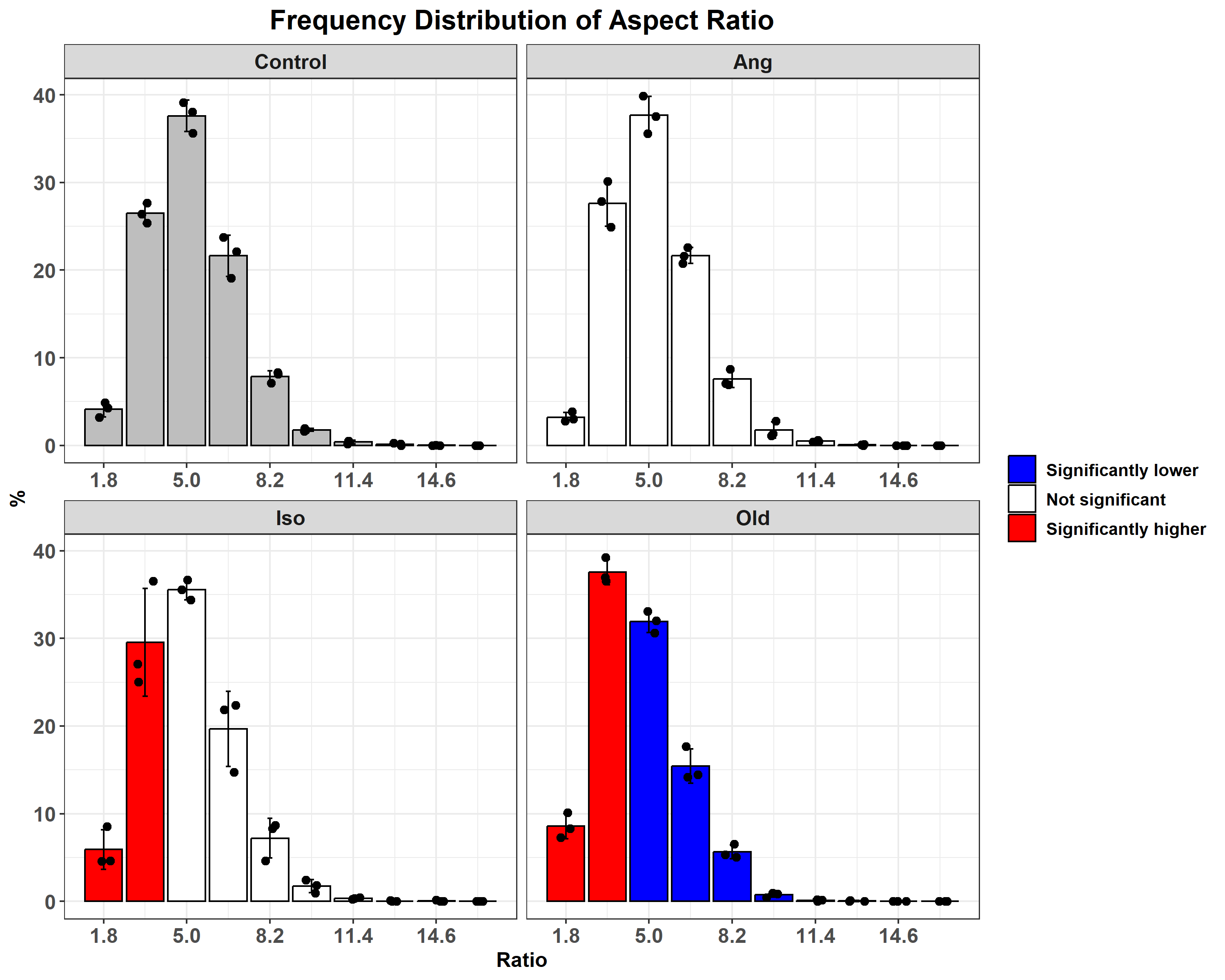

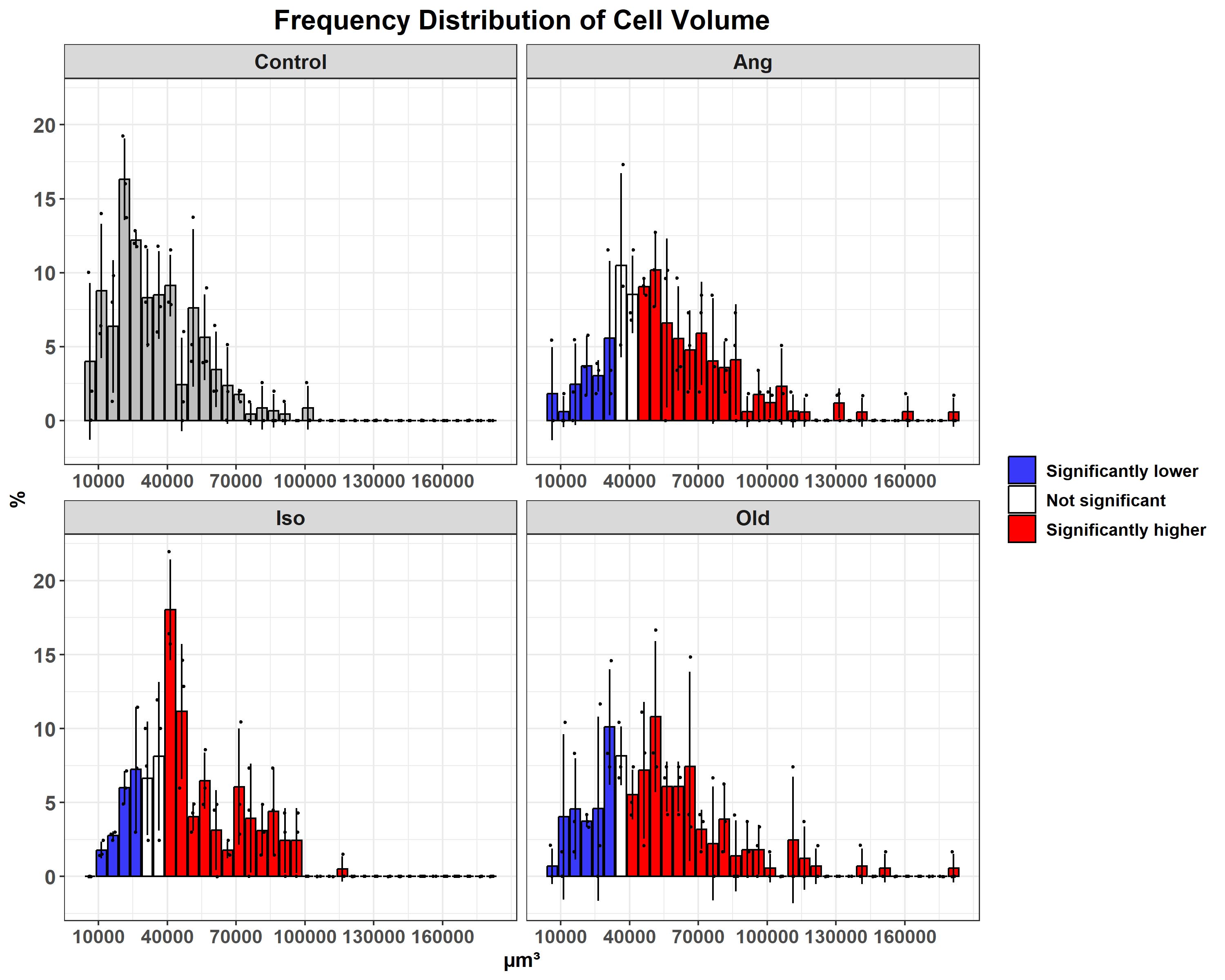

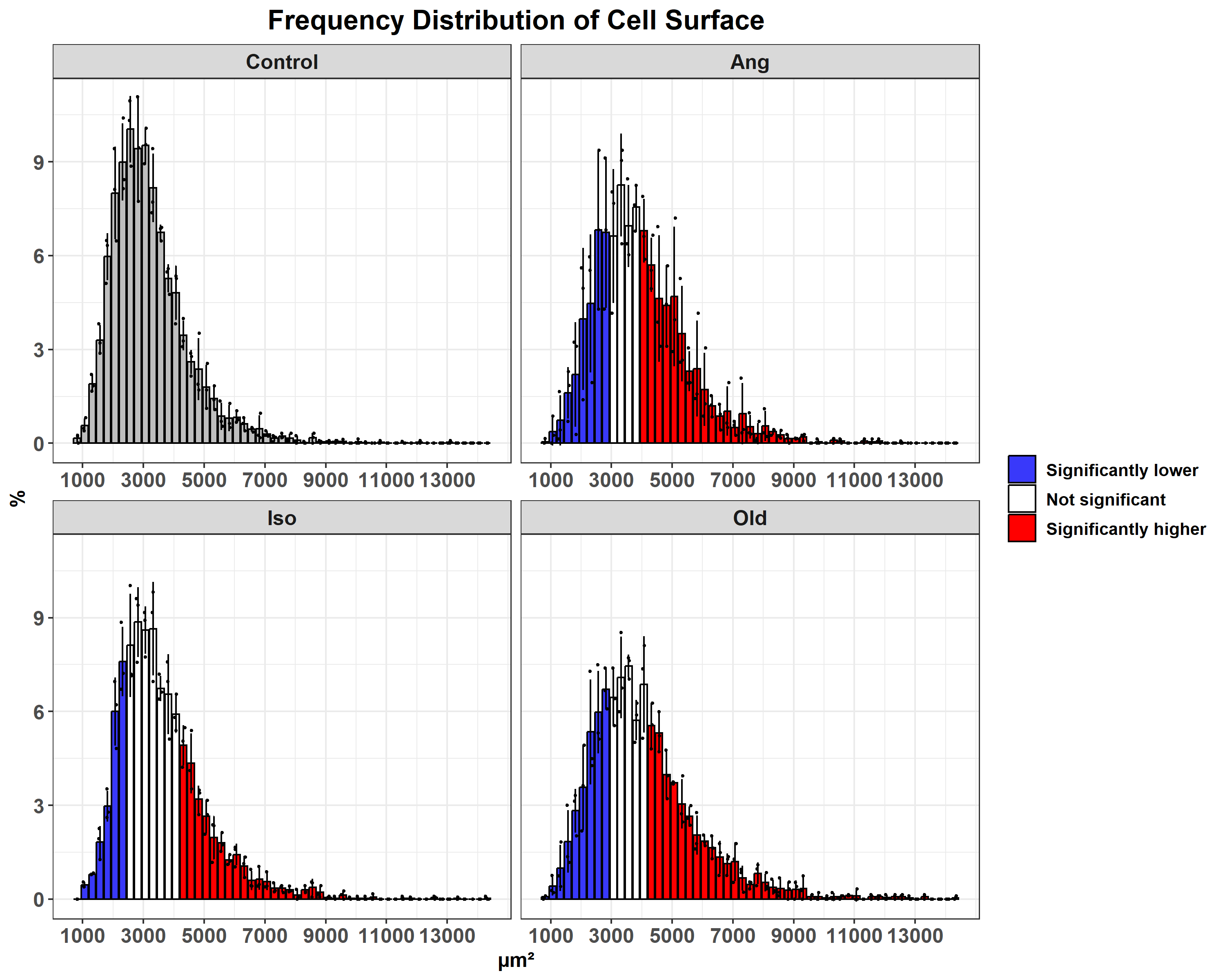


**B**

**C**

**D**

**E**

**A**

**Figure S7.** Frequency distribution of morphological features in four experimental groups.

Histograms display the percentage of cells distributed by **(A)** cell surface, **(B)** cell width, **(C)** cell length, **(D)** aspect ratio and **(E)** cell volume for the Control, Ang, Iso, and Old groups. The Control group (top left) is shown in grayscale. In the other groups, white bars represent no significant difference, blue bars indicate significantly lower frequencies, and red bars indicate significantly higher frequencies compared to Control (p adjusted <0.05). All statistical comparisons were performed using Fisher’s exact test. Data are expressed as mean ± SD.


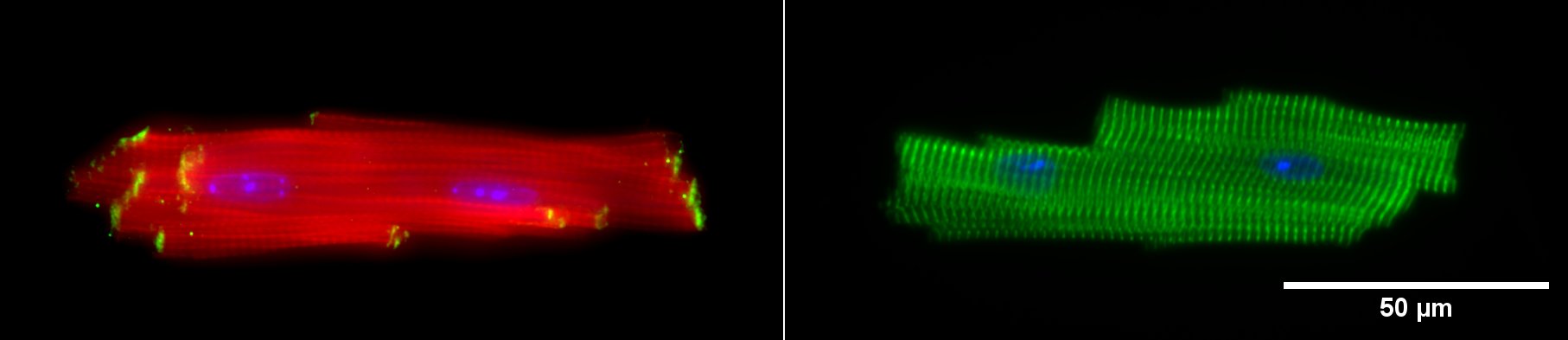


**A**

**B**

Figure S8. Cardiomyocytes after 4 months of freezing period in BamBanker™ at -80^o^C, stained for **(A)** Phalloidin (red), pan-cadherin (green) and Hoechst (blue); or **(B)** with alpha-actinin (green) and Hoechst (blue).

**Table S1.** Q-PCR primers sequences.

| **Primers** | **Forward 5’ -** | **Reverse 5’ -** |
| --- | --- | --- |
| Vimentin | 5'-TGGTTGACACCCACTCAAAA-3' | 5'-CCACCAAGTGTGTGCAATTT-3' |
| FAP | 5'-CACCTGATCGGCAATTTGTG-3' | 5'-CCCATTCTGAAGGTCGTAGATGT-3' |
| PECAM | 5'-GAGCCCAATCACGTTTCAGT-3' | 5’-TGTCCTTCCTGCTTCTTGCT-3' |
| SM22 | 5'-GGCTCAGAGCTTCAGAGGAA-3' | 5'-CTCTCCTTCTGCCAAACCAG-34 |
| ANF | 5′-GCCGCACTTAGCTCCCTCCCCGAG-3′ | 5′-GTACCGGAAGCTGTTGCAGCCTAG-3′ |
| BNP | 5'-CACCGCTGGGAGGTCACTCCT-3' | 5'-GCTCCAGCAGCTTCTGCATCT-3' |
| α-MHC | 5′-ACCGTCTGGACGAGGCAGAGCAGA-3′ | 5′-CGTCGTGCATCTTCTTGGCACCAA-3′ |
| β-MHC | 5′-CAGCACCGTCTGGACGAGGCAGAG-3′ | 5′-ATTCAGGCCCTTGGCACCAATGTC-3′ |
| ACTA1 | 5′-CTGAGCGCAAGTACTCAGTGTGGA-3′ | 5′-TTCCAAAAACAGGCGCCGGCTGCA-3′ |
| COL1α1 | 5'-GAGAGGTGAACAAGGTCCCG-3' | 5'-AAACCTCTCTCGCCTCTTGC-3' |
| COL3α1 | 5'-TGACTGTCCCACGTAAGCAC-3' | 5'-GAGGGCCATAGCTGAACTGA-3' |
| β-Actin | 5’-GATCTGGCACCACACCTTCT-3’ | 5’-GGGGTGTTGAAGGTCTCAAA-3’ |
| C-Actin | 5′-GAGACTCTCTTCCAGCCCTCTTTC-3′ | 5′-TCAGAAGCACTTGCGGCGGACAAT-3′ |
| HPRT | 5'-AGGACCTCTCGAAGTGT-3' | 5'-ATTCAAATCCCTGAAGTACTCAT |
| GAPDH | 5'-AACTTTGGCATTGTGGAAGG-3' | 5'-GGATGCAGGGATGATGTTCT-3' |
| HMBS | 5'-AGGTCCCTGTTCAGCAAGAA-3' | 5'-TGGGCTCCTCTTGGAATGTT-3' |
| 18S | 5'-CGCCGCTAGAGGTGAAATTC-3' | 5'-TCTTGGCAAATGCTTTCGC-3' |
| B2M | 5'-CACACTGAATTCACCCCCAC-3' | 5'-GTCTCGATCCCAGTAGACGG-3' |
| MYH14 | 5'-acttgagagcgtgtccacag-3' | 5'-agttcctgggtgtcatgcag-3' |
| GATA4 | 5'-TCCCAGGCCTCTTGCAATGCGGAA-3' | 5'-GCGGTGATTATGTCCCCATGACTG-3' |
| SRF | 5'-CTCCGCCCCGCTCAGACCCCACCACAGA-3' | 5'-CAGGTAGTTGGTGATGGGGAAGGA-3' |

**Supplemental Method 1:** Sarcomere length measurement


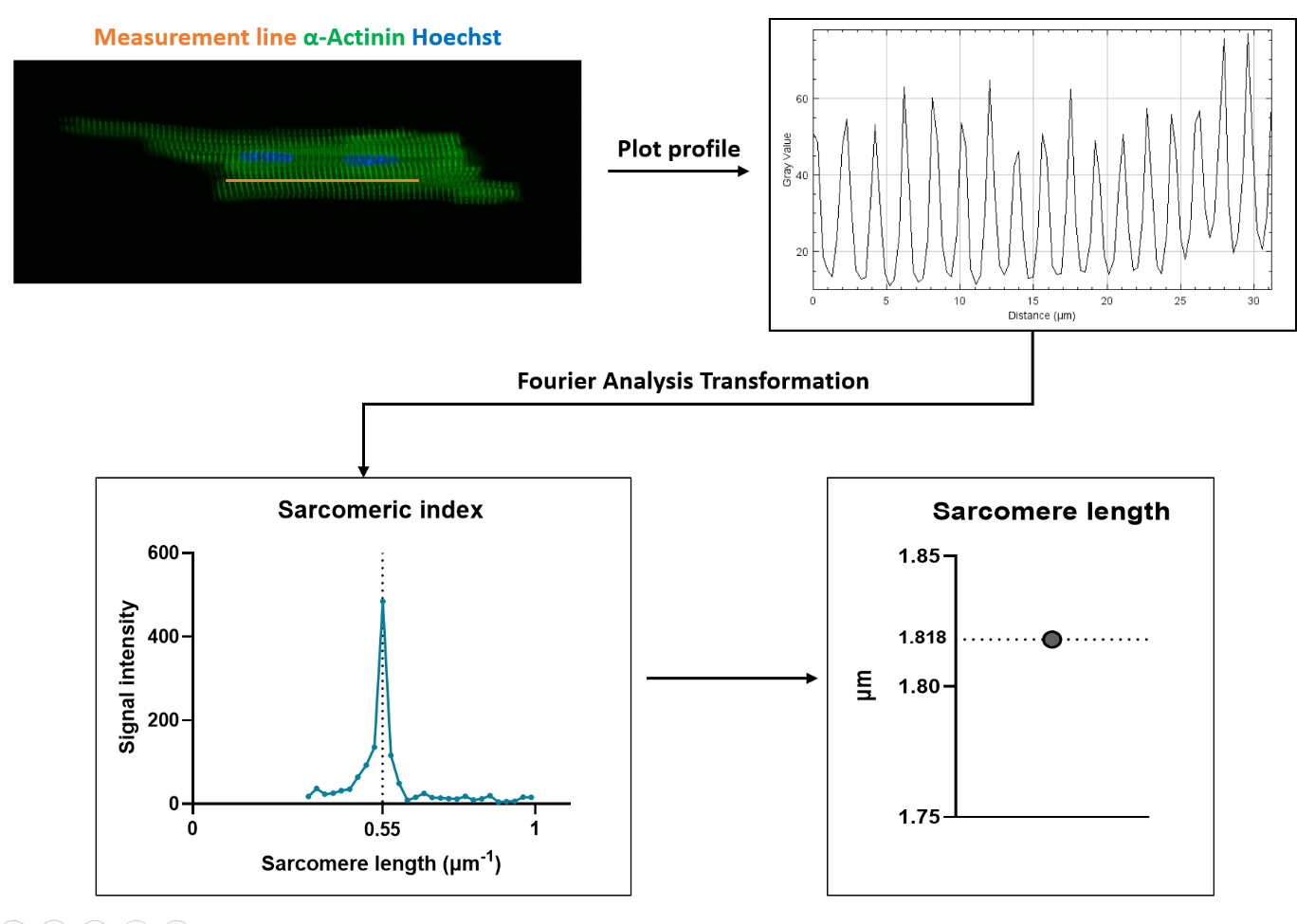
**Figure SM1.** The workflow for sarcomere length measurement.

The fluorescence images of cardiomyocytes stained for α-actinin were imported into ImageJ software. A measurement line was drawn across the stained cells to capture the repeating sarcomere pattern. The fluorescence signal along this line was measured and plotted for an illustration graph. The periodic peaks correspond to the locations of the Z-lines, which are rich in α-actinin. The plot profile was then analyzed using Fourier analysis, a method that transforms the spatial data (graph) into frequency data (graph). The peak indicates the most frequent repeating pattern, which corresponds to the average length of a sarcomere measured in the reciprocal of length (µm^-1^). Finally, this information was used to calculate the actual length (µm) of the sarcomeres.
