# Supplementary material for: Distinct morphometric features of cardiomyocytes isolated from mouse hypertrophy models: An ImageJ analysis combined with machine learning algorithms
Source: Physiol Rep. 2025 Jun 19;13(12):e70425. doi: 10.14814/phy2.70425 (PMC12179408; doi:10.14814/phy2.70425)
Supplement: Supplementary file 2 — Video S1. [file PHY2-13-e70425-s002.zip › VideoS1_caption.docx]

Video S1. Three-dimensional visualization of isolated cardiomyocytes from four experimental groups: Control, Ang, Iso, and Old. Cardiomyocytes were stained with Phalloidin (red), pan-Cadherin (green), and Hoechst (blue), and imaged using confocal microscopy. The X, Y, and Z axes represent spatial dimensions in micrometers. The video presents a 360-degree rotation of composite 3D images, illustrating the spatial organization, morphology, and structural differences among cardiomyocytes from each group. This provides a comparative visual analysis of cardiomyocyte dimensions and architectural remodeling induced by hypertrophic stimuli and aging.
